# Towards Healthy and Sustainable Diets: Understanding Food Consumption Trends in the EU

**DOI:** 10.3390/foods14162798

**Published:** 2025-08-12

**Authors:** Fabrizio Biganzoli, Carla Caldeira, Joana Dias, Valeria De Laurentiis, Joao Leite, Jan Wollgast, Serenella Sala

**Affiliations:** 1 UniSystems, Via Michelangelo Buonarroti 39, 20145 Milano, Italy; f.biganz@gmail.com; 2 European Commission, Joint Research Centre, Via Enrico Fermi 2749, 21027 Ispra, Italy; caldeira.carla@gmail.com (C.C.); joana.dias@ec.europa.eu (J.D.); valeria.de-laurentiis@ec.europa.eu (V.D.L.); joao.leite@ec.europa.eu (J.L.); jan.wollgast@ec.europa.eu (J.W.)

**Keywords:** food consumption, dietary trends, healthy eating, sustainable diets, cluster analysis

## Abstract

The assessment of food system sustainability requires a profound understanding of the evolution of food production and consumption. Monitoring the transition towards healthier and sustainable diets is crucial for supporting future interventions. This study explores market sales data as an input to investigate and compare current dietary trends in the European Union Member States over 14 years. By analysing consumption trends of major food product categories, including animal-based and plant-based foods, we identified two distinct clusters of countries with opposite dietary patterns. Our analysis explored not only major food product categories essential for healthy living but also superfluous foods (i.e., discretionary) to provide a broader understanding of dietary habits. In particular, our results show that countries reducing consumption of animal-based foods also exhibit a reduction in consumption of discretionary products, such as alcoholic beverages and soft drinks, highlighting opportunities for synergies between environmental and health policies. This research provides valuable insights for policymakers and stakeholders aiming to promote the uptake of healthy and sustainable diets and supports the development of targeted strategies to support the transition towards more environmentally friendly and healthy food systems.

## 1. Introduction

Sustainable food systems imply a transition to healthy and sustainable diets, which promote all dimensions of individuals’ health, reduce environmental pressure and impact, are accessible, affordable, safe, and equitable, and are culturally acceptable [1]. These diets predominantly consist of plant-based foods, abundant in fruits and vegetables, legumes, nuts, and wholegrains, and have low-to-moderate amounts of animal foods. However, in developed countries, average intakes of calorie-rich food, red meat, sugars, salt, and fats continue to exceed recommendations, while consumption of whole-grain cereals, fruit and vegetables, legumes, and nuts is insufficient. This is the case, for example, of the European Union (EU) [2], where the Farm to Fork strategy (F2F) aims at enabling a transition to fair, healthy, and sustainable food systems [3].

However, to design effective interventions for such a transition, it is essential to understand trends and the evolution of food consumption patterns, which is the main goal of this study. This will also allow assessing progress in the transition towards healthy and sustainable diets.

A transition towards healthier, plant-based diets would likely result in a better alignment with current dietary guidelines and sustainability goals [4]. This would entail a significant reduction in the consumption of animal-based food, particularly red and processed meat, while increasing legume, nut, fruit, and vegetable consumption [5]. Replacing animal products with plant-based alternatives is recommended by intergovernmental organizations [6] and widely integrated in national Food-Based Dietary Guidelines (FBDGs) in the EU [7,8]. The F2F strategy remarks that aligning to dietary guidelines would reduce the environmental footprint of food systems [9]. A study on the consumption footprint of the EU food system unveiled that animal-based products are driving the EU food-related impacts across 16 different environmental impact categories [10,11]. Particularly, current levels of meat consumption may result in unsustainable impacts on the health of consumers and of the environment [5].

Nevertheless, adopting plant-based diets is not necessarily healthy [12]. For instance, higher consumption of discretionary foods, including sugar-sweetened beverages, sweets and desserts, or refined grains, has been linked to unhealthier plant-based dietary patterns [13,14]. Discretionary foods are nutritionally and environmentally superfluous [15]. Excessive consumption of discretionary foods is associated with a higher risk of developing obesity and non-communicable diseases [16,17] and it is responsible for more than one-third of diet-related environmental impacts [15].

In addition, dietary shifts may cause changes in food waste generation patterns, both in terms of the most wasted products and where in the food supply chain (FSC) waste is generated. Caldeira et al. [18] observed that the FSC phase generating the largest share of food waste varies depending on the food type, with the most contributing phases for fruit and vegetables being primary production and consumption. De Laurentiis et al. [19] reported that in the EU fresh fruit and vegetables contributed to almost 50% of household food waste. Consumers’ preferences in respect to fresh and processed fruit and vegetables may shift the production of most food waste from the consumption to the manufacturing stage of the supply chain, requiring different measures and creating different opportunities for waste prevention.

This study aims at understanding the evolution of the EU food system in relation to food consumption patterns. According to Rippin et al. [20], dietary surveys at the national level are important to inform about dietary changes at the population level based on declared food consumption. However, the same authors pointed to significant limitations of dietary surveys for monitoring purposes due to the lack of methodological harmonization and the fragmented implementation across the EU. Since 2009, the European Food Safety Authority (EFSA) has been developing a harmonised methodology for the collection of harmonised food consumption data [21]. However, harmonised data require costly, intense surveys scarcely implemented in all countries and across population groups. FAO food balance sheets [22] are often used to monitor dietary trends by monitoring the supply of food commodities and supporting the development of national policies, but data is not adequate to inform on changes in the nutritional quality of the food choices.

Market-based sales data are regularly updated, and they are often provided in a harmonised way across countries. Despite not fully representing food consumption (achievable only by monitoring people’s daily consumption), sales data can support consumption estimation at the individual level.

The aim of this study was to analyse dietary trends across the 27 EU countries in the past decade and identify the gap between current trends and healthier and sustainable diets. For this we explored the usefulness of using market sales data.

Since Europe is not considered particularly homogeneous regarding food culture [23], we used cluster analysis to identify Member States (MSs) with similar trends and to better compare trends in different EU countries.

## 2. Materials and Methods

The assessment of the evolution of food consumption based on market-based sales data followed a method articulated in two steps: (a) the selection of product categories considered and data sources (Section 2.1); and (b) the trend and cluster analysis (Section 2.2). This is complemented by an overview of the available recommendations for healthy and sustainable diets (Section 2.3) to compare the trends against these recommendations.

### 2.1. Selection of Product Categories and Data Source

In this study, product-level data were obtained from Euromonitor International, which is a business intelligence provider of product sales data and market outlook of the food and beverage sectors, including fresh food, packaged food, alcoholic drinks, and soft drinks [24]. Annual data representing most of the retail market on food and beverages for 27 MSs from 2008 to 2021 were retrieved in November 2022. Approximately 66,990 sales data points were extracted for 120 food items, aggregated in 30 product categories (Table 1). Overall, trends could be estimated based on 11,340 data points (11,340 = 30 product categories * 27 MSs * 14 years).

The selection of the major food product categories reflects references for healthy and sustainable diets, including key groups in national food-based dietary guidelines and the planetary healthy diet [5]. This categorisation aimed to capture certain trends among food products central in current dietary transition, such as plant-based alternatives for milk and meat (Table 1). The selection consists of products from animal origin (red meat, poultry, fish, milk, cheese, yoghurt, and eggs), largely consumed plant products (fruits, vegetables, starchy vegetables, and cereals), plant-sourced proteins (nuts and legumes) and commercially available plant-based alternatives for meat and milk. The product’s disaggregation level reflects different nutritional and environmental implications. Despite the importance of wholemeal cereals in diets [5,8], whole and refined grains could not be monitored separately because these were lacking in the data source.

Consumption trends of these main food product categories were explored by means of cluster analysis to capture similarities among countries. Moreover, two additional groups of product categories were further analysed to observe consumers’ habits: discretionary foods (9 product categories) and fresh and processed fruit, vegetables, and starchy vegetables (6 product categories).

The first includes foods and drinks scarce in nutritional values and often high in saturated fats, sugars, salts, and/or alcohol [25]. Such energy-dense and nutrient-poor products affect the healthiness of dietary patterns [15] and they are aggressively advertised to consumers, and in high-income countries they gradually substituted healthy fresh foods [26]. Among the available discretionary products (i.e., alcoholic drinks, soft drinks, juice, frozen desserts, confectionery, savoury snacks, cakes, pastries, and sweet biscuits), fruit juice was included for its high free sugar content and because most FBDGs recommend limiting its consumption [7].

The second group focuses on fresh and processed fruit and vegetables, with starchy vegetables being often the most wasted products, with the largest share of food waste in the EU generated during consumption [27]. It investigates consumers’ purchasing trends towards these food products, relevant for its implication on food waste generation in different food supply chain phases.

### 2.2. Trend and Cluster Analysis

This section describes the procedure used to calculate trends (Section 2.2.1), and the clustering methods (Section 2.2.2). All analyses were performed using R v4.1.2 [28].

#### 2.2.1. Consumption Evolution over Time (2008–2021)

Sales per capita were evaluated in grams or millilitres per person^−1^ day^−1^. Product category sales are calculated as cumulative sales of all food items (e.g., apples, bananas, etc.) representative of a category (e.g., fruit), as in Table 1. Data availability varies for different years, countries, and products. The largest data gaps are observed for meat and milk alternatives, for which time series often start later than 2008.

Trends were calculated with robust linear regression [29] of market data normalised to the first year available (i.e., 2008). It represents the average variation (increase or decrease) in percentage points compared with the first year of the time series. Normalisation is necessary as sales amounts may vary significantly for products and countries. For instance, the market size for meat is two orders of magnitude higher than its plant-based alternatives.

Robust linear regression was performed using the “rlm” function in the MASS R package v7.3 [30]. Robust regression was chosen as it minimises the influence of potential outliers; no data points were excluded. An example of its application is reported in Supplementary Materials (SM1—Robust Linear Regression) [29,31]. Only one time series was excluded because it consisted of only 3 data points (Greece–Meat alternatives). Despite some food items for certain countries being modelled (instead of measured) in the data source, no data were excluded, but instead a sensitivity analysis was performed to ensure that modelled data would not affect the results (SM2—Cluster Analysis details) [32,33,34,35,36].

#### 2.2.2. Cluster Analysis

Clustering is the process of partitioning data into subsets [37]. Each cluster is defined as a collection of objects similar enough to each other and dissimilar to objects in other clusters [38]. Trend patterns among MSs were recognised by means of two approaches: hierarchical and k-means. Consensus between methods reveals more robust patterns.

Hierarchical approaches produce a plain tree (dendrogram) connecting objects based on their similarity; clusters are defined successively [39]. K-means assigns observations to a pre-defined number of clusters, aiming at optimizing intra-group homogeneity [40].

Supplementary Materials (SM2—Cluster Analysis details) detail the selection of the optimal number of clusters, the study of underlying relevant food consumption trends using principal component analysis (PCA), and sensitivity analysis to assess the robustness of results.

Lastly, a time series representative for each cluster was calculated as the average of consumption weighted for the population of each MS within the cluster. These profiles are available in Supplementary Materials (SM3—Clusters’ profile).

### 2.3. Recommendations for Healthy and Sustainable Diets

Recommendations for healthy and sustainable eating have been derived from multiple sources. FBDGs [7] act as science-based recommendations for consumer information and as a framework for a country’s nutrition policies, as issued by public health authorities. Historically, FBDGs have focused mostly on health promotion. However, with rising concerns for the environmental impact of diets, more countries have included the sustainability dimension in their FBDGs [41]. This could vary from encouraging consumption of seasonal products to gradually directing people to consume more plant-sourced proteins [7]. Indeed, the Nordic Council of Ministers has recently launched the new Nordic Nutrition Recommendations 2023 (NNR) [42], which includes health aspects and environmental concerns in all food groups. Adding to this picture, the EAT-Lancet Commission [5] clearly defined scientific recommendations for healthy eating within planetary boundaries.

These recommendations (summarised in Table 2) were compared against the consumption estimates and trends for each food product category and for each cluster. The consumption estimates were calculated as average consumption in MSs assigned to the cluster, weighted by their population.

## 3. Results

The results of the study are presented as follows: the consumption trends and the main drivers for MSs clustering, based on the 15 major food product categories (Section 3.1); consumption trends of discretionary foods (Section 3.2) and of fresh and processed foods (Section 3.3). Recommendations from FBDGs, NNR, and EAT-Lancet, as well as the consumption estimates calculated for each cluster, were collected in Section 3.4.

### 3.1. Cluster Analysis for Major Food Product Categories

**Figure 1:**
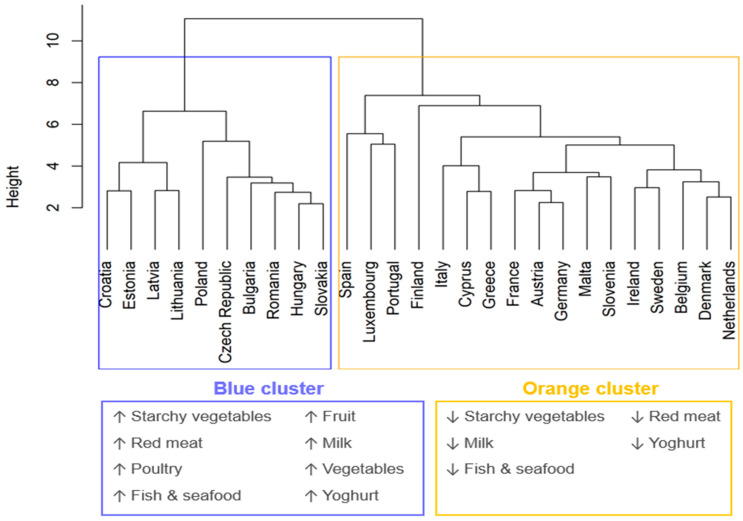
Dendrogram with clusters from hierarchical method based on the 15 major food product categories (Table 1) and relevant trends for main food groups responsible for the clusters division. Upward and downward arrows mean increasing and decreasing trends, respectively.

The two clustering methods used (i.e., hierarchical and k-means) produced the same partition of MSs. Figure 1 illustrates the dendrogram produced by the hierarchical method and lists the relevant trends responsible for cluster partitioning, identified with PCA (SM2—Cluster Analysis details). Colours (blue and orange) identify the clusters’ names. The *y*-axis measures the distance between countries.

**Figure 2:**
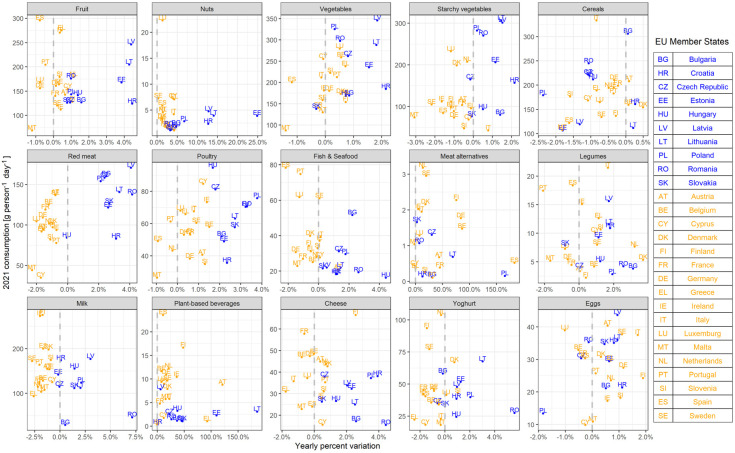
Consumption in 2021 (g person^−1^ day^−1^) and percentage of yearly variation (average value calculated over the range 2008–2021), at MSs level.

Figure 2 illustrates per capita consumption in 2021 (*y*-axis) and trends (*x*-axis) expressed as average yearly variation. The vertical dashed line separates increasing (on the right) from decreasing trends (on the left). For instance, for meat alternatives, the Netherlands lies in the upper left corner of the plot close to the dashed line, meaning high per capita consumption and a small increase over time, whereas Spain is in the bottom right corner, indicating low per capita consumption and a large increase. 

Figure 2 shows great variability in both consumption and trends among MSs. Clusters are well separated within the plot area into several product categories, with milk, red meat, and poultry showing the clearest division. Consumers in MSs in the blue cluster are increasing their consumption of animal products (red meat, fish and seafood, milk, cheese) from 2008 to 2021, whereas consumers in countries in the orange cluster show decreasing consumption trends (on average −1% per year). Poultry consumption is growing in most MSs, but at a slower rate (+0.5%) for MSs of the orange cluster compared to the blue cluster (+2.6%). Per capita consumption may vary significantly within clusters; for example, people in Austria consume four times more red meat than those in Cyprus.

Both clusters share similar trends for cereals, meat alternatives, legumes, and eggs. In particular, the consumption of nuts, meat alternatives, and plant-based beverages is increasing across all MSs, while cereal consumption is reducing except in a few countries, and together with fish and seafood, they are generally consumed more in the orange cluster. However, plant-based alternatives are consumed in much lower amounts compared to their animal-origin counterparts.

### 3.2. Consumption Trends of Discretionary Products

**Figure 3:**
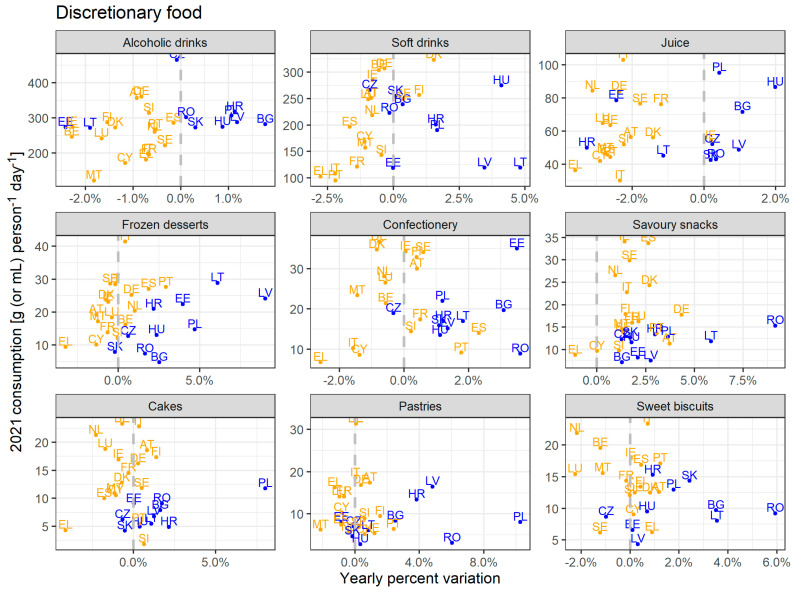
Consumption in 2021 (g person^−1^ day^−1^) and percentage of yearly variation of discretionary product (average value calculated over 13 years), at MSs level. Consumption of alcoholic drinks, soft drinks, and juice are expressed in mL person^−1^ day^−1^. Colours reflect clusters defined for major food groups (Figure 1).

Following the clustering of countries by dietary trends, market data allow comparing trends for foods perceived to be poor in nutritional value within the two clusters. This is particularly relevant, as a transition towards plant-based diets is not necessarily healthy [14]. Figure 3 illustrates consumption per capita in 2021 and trends are expressed in yearly variation.

In general, people in most MSs are consuming fewer alcoholic drinks (on average −0.6% per year) and juices (−1.5%) in the time series 2008–2021, mostly driven by the orange cluster, whereas consumption of savoury snacks is increasing across the EU, except for Greece. Trends for frozen desserts, confectionery, cakes, pastries, and sweet biscuits show great variability among MSs. Consumption trends in the blue cluster are increasing for frozen desserts (+3.3%), confectionery (+1.7%), savoury snacks (+3.2%), cakes (+1.5%), pastries (+2.7%), and sweet biscuits (+2.3%). Consumption of discretionary products per capita shows great variability among MSs, with generally higher values in the orange cluster.

### 3.3. Consumption Trends of Fresh and Processed Fruit, Vegetables and Starchy Vegetables

**Figure 4:**
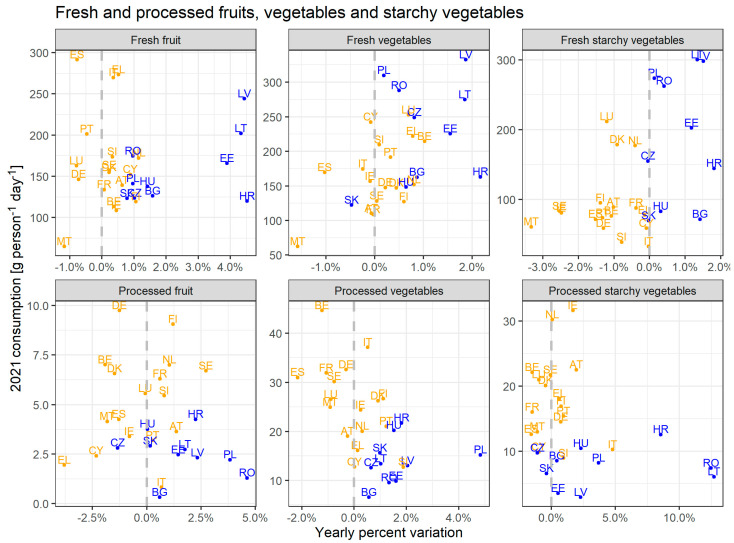
Consumption in 2021 (g person^−1^ day^−1^) and percentage of yearly variation of fresh and processed fruit, vegetables and starchy vegetables (average value calculated over 13 years), at MSs level. Colours reflect clusters defined for major food groups (Figure 1).

Differences in food consumption between fresh and processed fruit, vegetables, and starchy vegetables were explored in the clusters previously identified. Figure 4 shows consumption per capita in 2021 with trends expressed as yearly percentage variation.

In general, countries in the blue cluster show an increasing trend of consumption of fresh alternatives, more rapidly than those in the orange cluster. The blue cluster countries show increasing trends for both fresh and processed products, with the highest growth rates for fresh fruit (on average +2.4% per year) and processed starchy vegetables (+4.1%). Instead, the orange cluster presents high intra-cluster variability, except for a common reducing trend of consumption of fresh starchy vegetables (−1.1%). Several MSs assigned to the orange cluster (e.g., Italy, +5.0%) show an increase in consumption of processed starchy vegetables, which may indicate a substitution of the fresh product.

Consumption of fresh fruit, vegetables, and starchy vegetables is up to two orders of magnitude larger than their relatively processed counterparts. Consumption of fresh products is comparable between the two clusters, whereas processed foods are more consumed in MSs belonging to the orange cluster.

### 3.4. Dietary Recommendations and Consumption Estimates

Table 2 illustrates examples of healthy diet references and consumption recommendations from Food-Based Dietary Guidelines (FBDGs) [41], Nordic Nutrition Recommendations (NNR) [42], and the EAT-Lancet Commission [5]. Qualitative recommendations are reported in italics.

Table 3 reports the weighted average consumption per capita for clusters for the first (2008) and the last (2021) year in the time series. The magnitude of consumption changes (in Table 3) highlights what is behind the trends (Figure 2, Figure 3 and Figure 4). Between 2008 and 2021, the per capita consumption of major food groups increased for the blue cluster (i.e., fruits, vegetables, red meat, poultry, fish and seafood, milk, cheese, etc.). Whereas for the orange cluster, these food groups see a decrease in per capita consumption (except for poultry). The per capita consumption of fish and seafood and dairy remains notably higher in the orange cluster both in 2008 and in 2021, despite the opposite trends observed within clusters (i.e., increased consumption in the blue cluster and decreased consumption in the orange cluster).

Additionally, the consumption of fruit, vegetables, and particularly legumes and nuts, falls below recommended levels in both clusters, while red meat intake exceeds dietary guidelines (Table 2). Among discretionary foods, alcoholic beverages and soft drinks—both recommended to be limited or avoided—continue to be significant contributors to overall consumption in both clusters.

## 4. Discussion

The present study used market sales data to support the investigation of food consumption trends towards healthier and more sustainable diets.

Two clusters of MSs with clear distinct dietary trends were identified. MSs in the orange cluster (i.e., Austria, Belgium, Cyprus, Denmark, Finland, France, Germany, Greece, Ireland, Italy, Luxembourg, Malta, Netherlands, Portugal, Slovakia, Spain, and Sweden) have been moving in the past decade towards lower intakes of red meat, milk, fish & seafood, and starchy vegetables. MSs in the blue cluster (i.e., Bulgaria, Croatia, Czech Republic, Estonia, Hungary, Latvia, Lithuania, Poland, Romania, Slovakia) show a clearly opposite trend for the same food groups. Additionally, the blue cluster showed a marked trend towards increased consumption of most product categories. Increasing consumption trends of meat and milk alternatives were observed in both clusters.

The geographical distinction between the clusters (particularly the blue one, groups of MSs from the Balkan and Baltic areas) indicates that common factors might be the driver of the contrasting trends. Possibly, increasing living standards observed in blue cluster countries in the last years can explain these differences [43]. Whitton and colleagues [44] have shown that meat consumption is directly related to changes in growth domestic product (GDP) and increased affordability of these foods in emerging economies. In line with our results, the same study also suggests that red meat consumption may have reached a peak in many higher-income countries, as observed in many central and western EU countries.

The different trends observed raise reflections on how dietary choices driven by economic factors might influence the achievement of environmental and health recommendations.

Meat and other animal protein sources

The current estimates in all EU countries indicate that red meat intake is well above all recommendations (Table 2). In particular, red meat consumption is on average higher than 100 g/d among countries in the orange cluster and even higher in the blue cluster.

If current levels of consumption of red meat remain well above dietary guidelines, and with rising trends in the blue cluster, this could lead to deleterious health effects [45,46] and unsustainable environmental and climate footprints [5,10]. Countries within the blue cluster show challenges in reversing current red meat consumption trends and might need targeted policies. In contrast, consumers in countries in the orange cluster may be already replacing some of the red and processed meat with other protein sources such as poultry, eggs, and, to a lesser extent, commercially available meat alternatives.

Poultry, preferably lean options and without skin, is recommended as a healthier option [7] and with a lower environmental impact [46] compared to red meat. Current projections indicate that both production and consumption in the EU are expected to grow between 2020 and 2030 [43]. Thus, poultry is expected to be a possible alternative to the replacement of red meat for many EU consumers in the upcoming years [44]. In this study, poultry consumption increased in the past decade in almost all MSs, and it is being consumed in high quantities. Most MSs fall between the two recommendations, the FBDGs and the EAT-Lancet (Table 3), which means poultry consumption should not increase beyond current levels to keep consumption within planetary boundaries.

Whilst fish may also represent an alternative source of protein, its consumption increased only in the blue cluster. Fish consumption is related to local fishing cultures: not surprisingly, Spain and Portugal (with large Exclusive Economic Zones) are placed in the highest consumption levels. In this study, all countries consuming above 64 g/day are in a decreasing trend, while the remaining ones are between both recommendations, the FBDGs and the EAT-Lancet (Table 2). It is important to note that recommendations for fish consumption differ between guidelines focused on health and those prioritizing sustainability, hindering a clear analysis of dietary transitions. Although the consumption of fish has a significantly lower climate impact than red meat and lower than poultry [47], increasing fish consumption above the recommendations is likely to impair the sustainability of fish species. FAO reports that the percentage of stocks fished at unsustainable levels has tripled compared to the 1970s [48]. In this light, aquaculture may play a key role in satisfying fish demand. However, Salin and Arome Ataguba [49] claim that aquaculture governance and planning must improve substantially at local, national, and international levels to ensure sustainable growth of this practice.

Milk and dairy are considered a source of high-quality animal protein and micronutrients. The orange cluster shows a reduction in milk consumption, with less clearly visible patterns for cheese and yogurt. Milk production generates most of the environmental impact associated with dairy products [50], with the most milk-intense products, such as butter and cheese (and to a lesser extent yoghurt), performing worse. Average consumption of yoghurt in 2021 is higher than cheese in both clusters. However, in the orange cluster yoghurt reduced by 15%, while cheese reduced by 5% in the period analysed. This is aligned with general trends in the presence of people moving towards a more flexitarian diet, either for health or environmental reasons [51]. After quitting or reducing the consumption of meat, milk and yogurt follow, and finally cheese is the last one people give up. Indeed, Docherty and Jasper [52] observed that cheese is the animal product vegetarians are most attached to, as the further the product is from its animal origin, the more vegetarians are willing to consume it. This could explain the recent reduction in consumption of milk and increase in consumed cheese trends in Western Europe [52]. On the contrary, people living in the blue cluster are rapidly increasing both cheese (+31%) and yoghurt (+21%) consumption, which could result from the same economic factors outlined above for meat consumption. On average, the consumption of all dairy products combined in many MSs falls near the recommendations (Table 2).

To reduce the food consumption environmental footprint, yoghurt consumption should be preferred to cheese. Alternatively, Carlsson Kanyama et al. [53] recommend a dietary change towards plant-based dairy alternatives for their lower emissions relevant for climate change, acidification, eutrophication, and ozone depletion, as well as their better performance on resource use. Apart from plant-based alternatives to milk, other plant-based alternatives to dairy and dairy products still represent a niche in the food market, and data are scarcely available.

Milk and meat alternatives show better environmental performance compared with their animal-based counterparts [54,55]. Although their consumption is increasing (along with more options offered on the market), they are still consumed in much lower amounts than their animal-based counterparts. While gaining popularity across the EU, concerns have been raised regarding the nutritional profile of certain plant-based meat alternatives high in saturated fat and salt [56] and the potential nutritional impact of fully replacing milk with plant-based alternatives [57]. More restricted plant-based dietary patterns (e.g., vegetarian or vegan) have low environmental footprints and can reduce the risk of diseases, such as cardiovascular diseases, diabetes, and certain cancers, however, may increase the risk of microelement inadequacy if mismanaged [58]. Food innovation and reformulation could contribute to healthier and more sustainable food environments by providing more nutritious plant-based food products in the market, as long as it considers micronutrient composition, such as for iodine-fortified plant-based beverages [57].

Increasing availability of meat alternatives is a market response to a sought transition towards more plant-based diets. Indeed, Bryant [54] argued that plant-based alternatives are preferred over whole plant products for their better appeal, taste, and convenience, and Estell et al. [59] reported the choice of meat substitutes for a transition towards a plant-based diet in 22.1% of consumers. Trends of consumption of meat products were further compared with EU consumers’ attitudinal surveys (SM4—Comparison with attitudinal surveys). MSs where respondents reported a stronger attitude to “eat less meat” as one of the actions taken to reduce their contribution to climate change are effectively evolving towards a reduction in red meat consumption in favour of their plant-based alternatives, while in countries belonging to the blue cluster, a lower share of respondents declared such an attitude, corroborating the findings of this work. The integration between the analysis of market data and attitudinal surveys allows us to better understand the drivers of dietary shifts.

Plant-based protein sources

Legumes can provide good-quality plant-based protein, contribute to a healthy diet, and reduce environmental impacts and soil quality [60]. In this study, legume consumption across the EU is well below current dietary guidelines for the majority of the MSs (Table 2). The role of legumes should be better emphasised in dietary interventions to overcome existing cultural and societal impediments [61]. Röös et al. [62] calculated that replacing 50% of Swedish meat consumption with legumes would reduce climate change impacts by 20% and land use by 23%, without worsening the nutritional dietary aspects.

Nuts are also sources of plant-origin proteins and nutrient-dense food and therefore key components of sustainable diets [5]. Vanham et al. [63] estimate that global nut production should increase 7 to 11 times to satisfy the daily per capita intake recommended by EAT-Lancet, and they propose options, such as employing nut-specific water footprint benchmarks, to achieve this target in a water-sustainable way. Nuts consumption (evaluated just for unprocessed nuts) is increasing in all MSs, with a faster rate in MSs assigned to the blue cluster. It shows an evolution towards dietary recommendations (Table 2), though clearly very far from it (almost all MS are below 10 g/day). However, the environmental, nutritional, and social performance may vary significantly within the nuts group, with walnuts scoring best whilst cashews, hazelnuts, and chestnuts are the least preferable [64].

Fruit and vegetables

Fruit and vegetable consumption varies broadly across different countries, even within clusters. However, most countries are not reaching the recommendations (Table 2), but the majority have been increasing their vegetable consumption. MSs in the blue cluster present a faster increase in both fruit and vegetable consumption. In 2019, one-third of EU citizens reported not consuming any fruit or vegetables daily [65]. Lower prevalence of daily fruit consumption has even been reported among children aged 11–15 years [66]. Overall, these results indicate the need for more action to help citizens make healthier and more sustainable food choices.

Discretionary foods

Guidelines often recommend limiting, avoiding, or reducing the consumption of discretionary food (Table 2). Given their poor nutritional and environmental performance, interventions should also target discretionary products to achieve F2F strategy goals. Importantly, a transition towards more plant-based diets should align environmental and nutritional goals. Certain foods such as sugar-sweetened beverages, refined grains, and sweets may have lower environmental footprints but have been linked to poor health outcomes. From an environmental angle, Saxe et al. [67] observed that halving consumption of alcoholic beverages, hot drinks, and sweets would also result in lower greenhouse gas emissions, equal to reducing meat consumption by 30%. Common reduction trends of consumption of alcoholic and soft drinks are observed in almost all MSs in the orange cluster and in few MSs in the blue cluster. This could result from more health awareness or more policies targeting these products [68]. However, countries in the blue cluster register growing trends for many of these products. Similarly to increased meat consumption, this could be associated with economic growth [69].

In a study developed by the JRC [10] assessing the food consumption footprint, namely the life-cycle environmental impacts of food consumption of EU citizens using 46 representative products, chocolate’s contribution per kilo of product is higher than the one of red meat for water use, mineral resource depletion, and freshwater ecotoxicity impact categories. Life Cycle Assessment studies from Nilsson et al. [70] calculated that sweets (i.e., foam sweets and jelly sweets) have a higher environmental impact than savoury snacks and soft drinks. MSs progressively transitioning towards a more plant-based diet also show a reduced consumption of alcoholic and sugar-sweetened beverages, whereas consumption of other discretionary products, such as confectionery (which includes both chocolate and sweets), is increasing in some MSs.

Food waste

Aside from the potential health and environmental consequences associated with the consumption of certain products, another relevant aspect when assessing dietary shifts is how these can affect food waste generation. De Laurentiis et al. [19] argued that the unavoidable component of food waste (i.e., food inedible under normal circumstances, e.g., peels) represents a “waste floor” to be considered in monitoring food waste prevention policies. This inedible waste baseline may shift from the household to the manufacturing phase in response to a growing preference for products with a higher level of processing. This has important consequences from a circular economy perspective, as inedible food waste generated at the processing stage has a higher valorisation potential than at the household stage [19]. For instance, an increase in the generation of unavoidable food waste at manufacturing might take place in Poland and Romania due to an increase in the consumption of processed plant products. However, as these countries reported a modest increase in fresh product intake, the unavoidable food waste generated at the household level is expected to remain stable. The ongoing shift of inedible food waste should be properly accounted for in monitoring frameworks.

Strengths and limitations

Market-based sales data can provide a detailed picture regarding market dynamics at the retail level examining the high granularity of products and thus providing a good overview of consumption trends. It also allows for a cross-country comparison due to the systematic nature of data collection. In the context of monitoring the transition towards a healthier food offer to consumers, market sales data have been used as a complementing tool to monitor food consumption trends [71]. Understanding how observed trends are in line with nutritional and environmental recommendations may support better and more targeted decisions for policy making.

Despite not fully representing consumption, market sales data are considered a good proxy for it. A quantitative analysis to estimate the gap between markets’ sales data and consumption from dietary surveys could not be performed due to the lack of harmonization of dietary surveys across countries and the lack of yearly datapoints for each country. However, from a qualitative perspective, sales data are expected to slightly overestimate the consumption, neglecting the cooking and accounting for the waste at households and food services [72]. Although sales data are not equivalent to food consumption data surveys, results can be transferred from one to the other while acknowledging their limitations. For instance, the high attitude to eat less meat recorded in the Dutch sample is not only reflected in the consumption trends observed in this study, but it is also confirmed by a recent national food consumption survey [73], which identified that consumption of red and processed meat decreased by more than 20% over the period 2007–2010 to 2019–2021. Therefore, sales data can provide good insights into consumption patterns and trends in a less costly manner.

Our study would benefit from price data to be paired to the sales data presented, to correct cost analysis and consumption trends. However, we did not have access to this data and considered that despite this limitation, the trends observed at the country level would not change majorly.

## 5. Conclusions

This study identified two clusters of MSs with opposite dietary trends of key food categories, particularly for red meat and milk. This would need to be tackled differently by practical interventions, e.g., at a policy level and by education initiatives. Additionally, clusters identified a group of MSs where a dietary transition is likely ongoing.

This work highlights the importance of having a comprehensive vision of food trends across the EU for a better understanding of environmental and nutritional implications of dietary changes. It will be crucial to consistently monitor food consumption trends—market sales data offer harmonised and yearly updated data for monitoring purposes—and identify key dietary issues for the different MS in the EU. Indeed, this research provides valuable insights for policymakers and stakeholders aiming to promote the uptake of healthy and sustainable diets and supports the development of targeted strategies to support the transition towards more environmentally friendly and healthy food systems.

## Figures and Tables

**Table 1:** List of food items from the data source aggregated in each product category.

	Product Category	Food Items (as from the Data Source)
**Major food product categories**	Fruit	Apples; Banana; Cherries; Cranberries/Blueberries; Grapefruit/Pomelo; Grapes; Kiwi fruit; Lemon and limes; Oranges, tangerines and mandarins; Peaches/nectarines; Pears/quinces; Pineapple; Plum/sloes; Strawberries; Other fruits; Shelf stable fruit; Frozen fruit
	Vegetables	Cauliflowers and broccoli; Maize; Onion; Tomatoes; Other vegetables; Shelf stable tomatoes; Shelf stable vegetables; Frozen processed vegetables
	Starchy vegetables	Cassava; Potatoes; Sweet potatoes; Other starchy roots; Frozen processed potatoes
	Cereals	Bread; Noodles; Pasta; Rice
	Nuts	Almonds; Coconuts; Peanuts; Pistachio; Walnuts; Other nuts
	Red meat	Beef and veal; Lamb, mutton & goat; Pork; Shelf stable processed red meat; Chilled processed red meat; Frozen processed red meat
	Poultry	Poultry; Shelf stable processed poultry; Chilled processed poultry; Frozen processed poultry
	Fish and seafood	Fish; Crustaceans; Molluscs and cephalopods; Shelf stable seafood ^1^; Chilled processed seafood ^1^; Frozen processed seafood ^1^
	Meat alternatives	Tofu and derivates; Shelf stable meat and seafood ^1^ substitutes; Chilled processed meat and seafood ^1^ substitutes; Frozen processed meat and seafood ^1^ substitutes
	Legumes	Beans; Peas; Other pulses; Shelf stable beans
	Milk	Cow’s milk; Powder milk; Goat milk
	Cheese	Spreadable cheese; Processed cheese excl. Spreadable; Hard cheese; Soft cheese; Fromage frais and quark
	Yoghurt	Sour milk products; Yoghurt
	Plant-based beverages	Soy drinks; Other plant-based milk ^2^
	Eggs	Eggs
**Discretionary products**	Alcoholic drinks	Beer; Cider/Perry; RTDs; Spirits; Wine
	Soft drinks	Flavoured bottled water; Cola carbonates; Non-cola carbonates; Concentrates; Energy drinks; Sport drinks
	Juice	100% juice; Nectars (25–99% juice); Juice drinks (up to 24% juice); Coconut and other plant waters
	Frozen desserts	Frozen yoghurt; Ice cream
	Confectionery	Chocolate confectionery; Sugar confectionery
	Savoury snacks	Nuts, seeds and trail mixes; Salty snacks; Savoury biscuits; Popcorn; Pretzels; Other savoury snacks; Fruit snacks; Snack bars
	Cakes	Cakes
	Pastries	Pastries
	Sweet biscuits	Chocolate coated biscuits; Cookies; Filled biscuits; Plain biscuits; Wafers
**Fresh and processed fruit, vegetables and starchy vegetables**	Fresh fruit	Apples; Banana; Cherries; Cranberries/Blueberries; Grapefruit/Pomelo; Grapes; Kiwi fruit; Lemon and limes; Oranges, tangerines and mandarins; Peaches/nectarines; Pears/quinces; Pineapple; Plum/sloes; Strawberries; Other fruits
	Processed fruit	Shelf stable fruit; Frozen fruit
	Fresh vegetables	Cauliflowers and broccoli; Maize; Onion; Tomatoes; Other vegetables
	Processed vegetables	Shelf stable tomatoes; Shelf stable vegetables; Frozen processed vegetables
	Fresh starchy vegetables	Cassava; Potatoes; Sweet potatoes; Other starchy roots
	Processed starchy vegetables	Frozen processed potatoes

^1^: In the data source, fish are included in the “seafood” definition. ^2^: in accordance with EU Regulation 1308/2013 and the ECJ judgment C422/16 the term plant-based milk cannot be used in commerce in EU. However, it is kept in this table exclusively, as it was employed in the data source referenced in the study (Euromonitor), to ensure the integrity, traceability, and reproducibility of the study and its methodology.

**Table 2:** Consumption recommendations from Food-Based Dietary Guidelines (FBDGs), Nordic Nutrition Recommendations (NNR), and EAT-Lancet Commission. Qualitative recommendations in italics.

Food Groups	Sub-Groups	FBDGs *	NNR	EAT-Lancet
**Meat**		2–3 times/week Or <500 g/week (i.e., circa 71 g/d) ^¥^		
	Read meat	*Eat less red and processed meat*	<350 g/w (i.e., circa 50 g/d)	14 (0–28) g/d
	Poultry	*Prefer lean meat*	*As low as possible* *Consumption should not increase from current levels*	29 (0–58) g/d
**Eggs**		<5 unit/week (1 egg ~ 60 g) (i.e., circa 43 g/day) ^¥^	1 unit/day	13 (0–25) g/d
**Fish and seafood**		1–4 portions/week Or 450 g/week (i.e., circa 64 g/d) ^¥^ *With emphasis on omega-3-rich fish*	300–450 g/w At least 200 g/w of fatty fish	28 (0–100) g/d
**Legumes**		2–4 portions/week (~30–100 g/portion) (i.e., circa 57 g/d) ^¥^ *Can be alternative to meat*	*Should be included as a significant part of the regular dietary pattern*	75 (0–100) g/d
**Nuts**		1–2 portions/d Or 25–50 g/d *Should be unsalted*	20–30 g/d *Also include seeds*	50 (0–75) g/d
**Milk and dairy**			350–500 g/d *Low fat*	250 (0–500) g/d
	Milk	2–3 portions/d Or 200–250 mL/d *Or on average a glass of milk or yogurt*		
	Yogurt			
	Cheese	1–2 slices(portion)/d Or 50–60 g/d *Of lean cheese*		
**Fruit and vegetables**		3–7 portions/d 400–650 g/d *With a 2:1 ratio (V:F)*	>500–800 g/d *Excluding potatoes and pulses*	
	Fruits	2–4 portions/d 200–250 g/d		200 (100–300) g/d
	Vegetables	3–5 portions/d 300–400 g/d		300 (200–600) g/d
**Discretionary foods (sweets/deserts and snacks)**		<1–2 portion/w *Limit or avoid consumption*	*Limited consumption*	
	Sugar	<10% energy intake (ideally <5%) Or 25–50 g/d		31 (0–31) g/d
**Alcohol**		<1–3 drink/d Or <10–20 g/d *Limit or avoid consumption*	*No safe lower limit* *Abstinence advised for some groups*	

*: FBDGs in Europe represent an estimated average of the recommendations taking into consideration several European countries, which differ considerably in some food groups. ^¥^: Average derived from the weekly recommendations divided by 7 days.

**Table 3:** Consumption per capita per product category and cluster. Data are reported for 2008 and 2021, which are the first and last year of the time series, respectively.

	Blue Cluster (g or mL/Person/Day)		Orange Cluster (g or mL/Person/Day)	
Product category	2008	2021	2008	2021
**Fruit**	126.2	148.9	200.5	196.0
**Vegetables**	246.9	269.6	187.2	184.0
**Starchy vegetables**	218.0	219.3	99.3	89.7
**Cereals**	251.1	206.4	229.2	208.5
**Nuts**	1.1	2.3	3.0	3.7
**Red meat**	108.4	140.4	126.3	105.9
**Poultry**	55.7	72.2	49.1	52.0
**Fish and seafood**	21.9	26.6	47.2	40.0
**Meat alternatives**	0.4	0.7	0.4	1.4
**Legumes**	4.7	5.2	10.2	10.6
**Milk**	88.3	108.7	199.5	156.0
**Cheese**	23.1	30.2	45.7	43.3
**Yoghurt**	31.5	38.1	52.9	44.7
**Plant-based beverages**	0.4	1.7	3.7	10.2
**Eggs**	26.7	24.9	28.9	30.7
**Alcoholic drinks**	347.0	333.7	326.0	276.6
**Soft drinks**	206.9	215.8	218.5	199.5
**Juice**	74.8	71.1	86.0	62.8
**Frozen desserts**	9.4	12.9	22.1	23.0
**Confectionery**	14.8	17.6	22.4	22.2
**Savoury snacks**	8.8	12.7	16.0	20.9
**Cakes**	6.3	8.8	16.6	15.8
**Pastries**	4.5	6.6	14.9	14.1
**Sweet biscuits**	8.8	11.0	15.4	15.9
**Fresh fruit**	124.3	146.6	193.8	190.3
**Fresh vegetables**	236.2	255.8	153.6	153.0
**Fresh starchy vegetables**	211.4	210.9	83.5	74.0
**Processed fruit**	2.0	2.3	6.7	5.7
**Processed vegetables**	10.8	13.8	33.6	31.0
**Processed starchy vegetables**	6.7	8.3	15.8	15.8

## Data Availability

The original contributions presented in the study are included in the article/Supplementary Material, further inquiries can be directed to the corresponding author. Research data used in this study is protected by copyright and cannot be shared. Specifically, all source material cited as “Euromonitor International, 2022” throughout the article is © Euromonitor International Ltd. [2022] and provided without any warranties or representations about accuracy or completeness. Further sharing, disclosure, publication or making available Figure 2, Figure 3 and Figure 4 will require Euromonitor’s prior written consent. Euromonitor International Ltd. cannot be held liable for analysis or findings within this study and cannot be held liable for any reliance on such materials in any capacity, and any reliance is done at the user’s risk.

## Supplementary Materials

The following supporting information can be downloaded at: https://www.mdpi.com/2304-8158/14/16/2798/s1. SM1: Robust linear regression; SM2: Cluster analysis details; SM3: Clusters’ profiles; SM4: Comparison with attitudinal surveys.
