# Supplementary material for: Towards Healthy and Sustainable Diets: Understanding Food Consumption Trends in the EU
Source: Foods. 2025 Aug 12;14(16):2798. doi: 10.3390/foods14162798 (PMC12385934; doi:10.3390/foods14162798)
Supplement: Supplementary file 1 [file foods-14-02798-s001.zip › foods-3635696-supplementary.pdf]

Supplementary Materials

Towards healthy and sustainable diets: understanding food consumption trends in the EU

Fabrizio Biganzoli[1], Carla Caldeira[2], Joana Dias[2], Valeria De Laurentiis[2], Joao Leite[2], Jan Wollgast[2], Serenella Sala[2,*]

[1] UniSystems, Via Michelangelo Buonarroti 39, 20145 Milano, Italy

[2]European Commission, Joint Research Centre, Via Enrico Fermi 2749, 21027 Ispra, Italy

[*]Corresponding author E-mail contact: serenella.sala@ec.europa.eu

Table of Contents

1. Robust linear regression 2

2. Cluster analysis details 6

   Optimal number of clusters 6

   Clusters and most relevant food trends 6

   Sensitivity analysis 8

3. Clusters' profiles 11

   Major food groups 11

   Discretionary products 13

   Fresh and processed fruit, vegetables and starchy vegetables 15

4. Comparison with attitudinal surveys 16

References 17

1. Robust linear regression

In the current study, food consumption trends were evaluated by means of linear regression models. As a good practice, outliers are identified and discarded before interpolating the linear models. Outliers are suspicious data points which may compromise the quality of the regression. Outliers may be affected by sampling errors, and should always be double-checked so to not derive biased results. Outliers' removal, when data cleaning is not available, is fundamental when the model is used to infer causality between the two variables. In this study, the regression model is used to assess trends, therefore the presence of eventual outliers is less critical compared with models for inferring causality. However, outliers should be properly managed to avoid data skewedness and they may reduce the goodness of fit of the curve.
During the initial data exploration phase, it was observed a sales decrease in 2020 in almost all food categories, with partial recovery in 2021, as consequence of coronavirus crisis. Indeed, the analysis of the residuals (i.e., distance between observed and predicted values) identified that around 50% of potential outliers (i.e. exceeding 1.5 times the interquartile range) refer to years 2020 and 2021. Despite the fact that these data are influential in the regression, they cannot be excluded because they represent a correct observation of reality. These data points are real correct values which cannot be considered neither suspicious nor wrong. Robust linear regression (Li, 2006) was used to better deal with these data. Robust regression applies a weighted linear model, in which the influence of suspicious data is downscaled. Observations with small residuals receive a weight of 1 and the larger the residual the smaller the weight (Hubert, 2011).

The analysis of the time series for "Processed starchy vegetables" in Czech Republic is reported as example of the benefit of using the Robust Linear Regression.

Figure SM 1.1 illustrates the data points distribution in the example. The blue dashed line represents the linear model calculated including all data points, the angular coefficient (-0.16) is used to quantify the trend. In Figure SM 1.1, the data points show fluctuations around a constant decreasing trend. In 2020 the consumption drastically plummeted and in 2021 the consumption partially recovered.

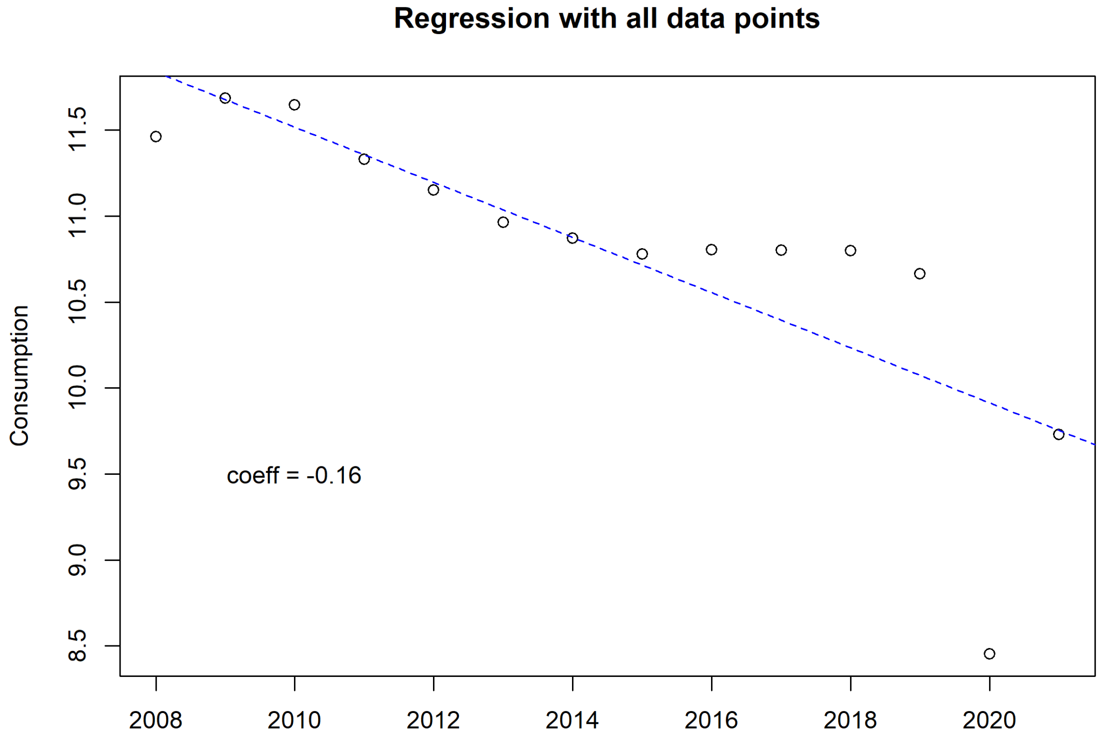

Figure SM 1.1. Visualisation of data points and linear regression model (blue dashed line).

Figure SM 1.2 confirms that the data point related to 2022 has a residual largely exceeding the residual normal distribution. The influence of this point was also confirmed by the analysis of Cook's distance. Data point for 2021 is not considered outlier despite being significantly lower than consumption values before 2020.

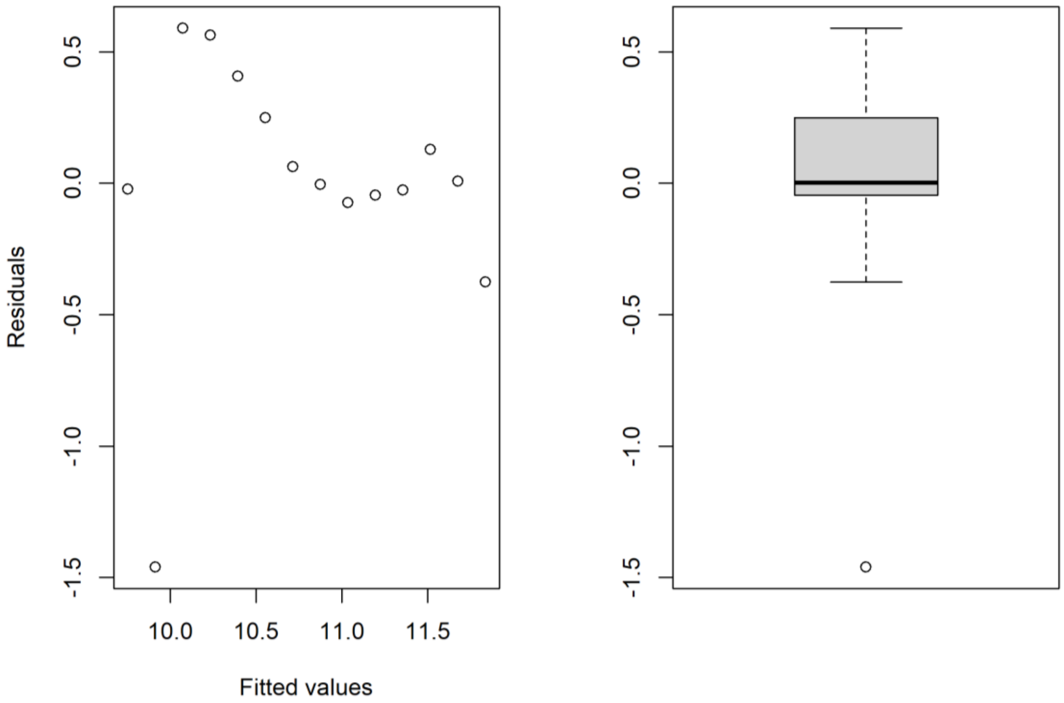

Figure SM 1.2. Distribution of residual and graphical representation of the data point largely exceeding the interquartile range.

Robust regression does not exclude this suspicious value, but downgrades its relevance (i.e., weight) in the regression. Figure SM 1.3 reports the regression curve generated with robust regression as well as the weights applied to each data point. Weights vary in the range 0 - 1. The regression coefficient is slightly lower compared to the one in Figure SM 1.1.

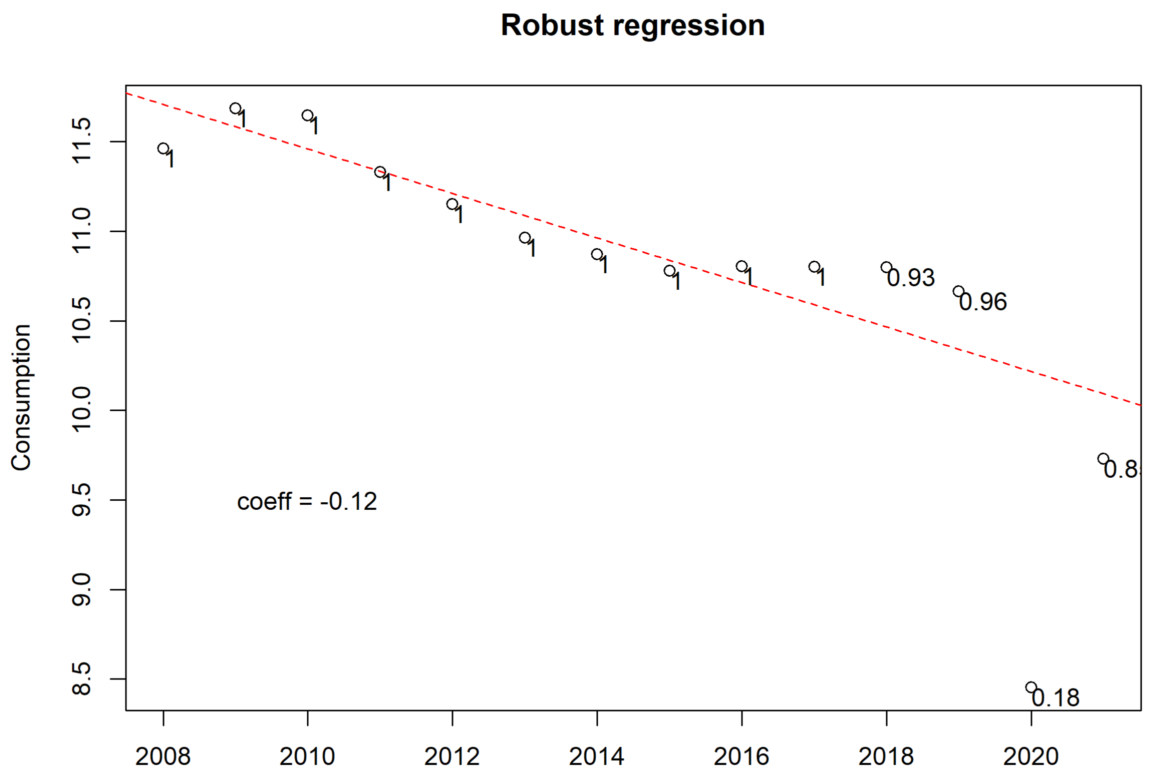

Figure SM 1.3. Robust regression and the weights applied.

The benefit of using robust linear regression could be evaluated in comparison with other models. Figure SM 1.4 compares the outcomes of ordinary linear regression with all data points (blue), robust linear regression (red), and linear regression excluding 2020 as considered outlier (green).

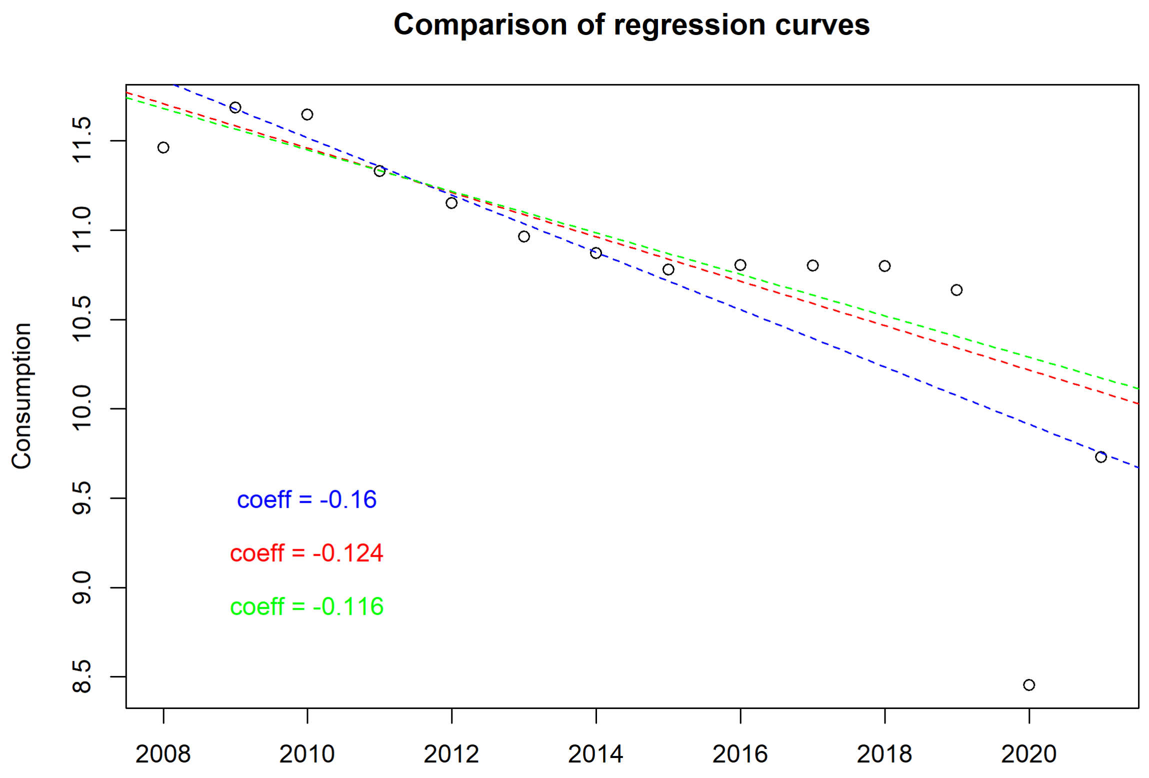

Figure SM 1.4. Comparison of linear regression using all available data points (blue), robust regression (red), and linear regression excluding 2020 (green).

The red and green lines are very similar. It confirms the beneficial effect of robust regression. It allows to estimate consumption trends (i) without being affected by suspicious data points, and at the same time (ii) to avoid excluding possibly trustworthy data points.

2. Cluster analysis details

Optimal number of clusters

The optimal number of clusters were defined by means of several available indexes. Three indexes were graphically consulted (elbow, silhouette, and gap statistic). The dashed line indicates the suggested number. In addition, the fourth sub-graph compares 30 indexes and counts the frequency of each proposed result (Charrad et al, 2014).

The "elbow" index (Thorndike, 1953) evaluates the intra-cluster distance among objects (WSS, Within-cluster Sum of Squares). The optimal number of clusters is defined as the "elbow" of the curve, which is the point where the slope significantly reduces. The "silhouette" index (Rousseeuw, 1987) measures the similarity inside a cluster. The higher the better. The "gap statistic" index (Tibshirani et al, 2001) compares the intra-cluster distance with the expected value from a null reference distribution. The higher the gap the better. As a general rule for all indexes, the lower the number of clusters the better, as long as it could provide a satisfactory interpretation of results.

The optimal number of clusters depends on the clustering method: different algorithms may perform differently on the same input datasets. In this study, all indexes agree that 2 is the optimal number for both clustering methods. Figure SM 2.1 illustrates the results of the optimal number of clusters analysis for k-means method. The results for hierarchical method are not visualised as produced the same outcomes.

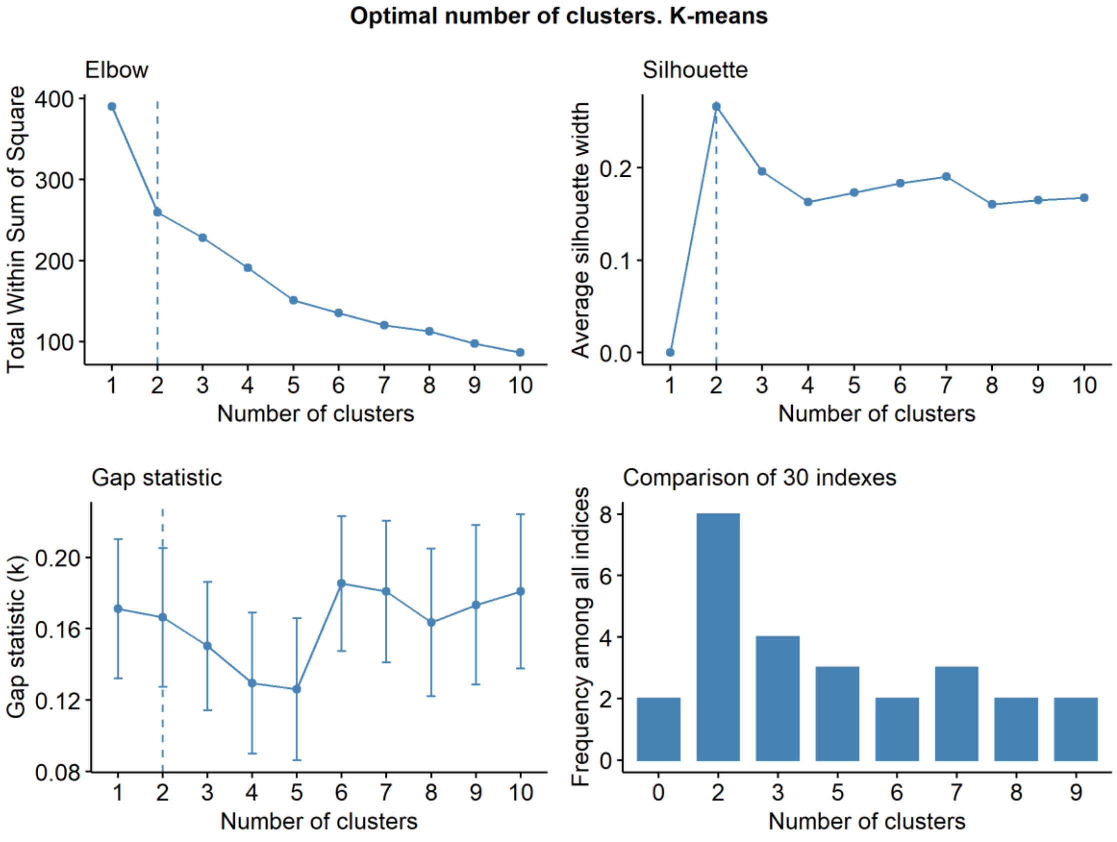

Figure SM 2.1. Optimal number of clusters for k-means clustering method.

Clusters and most relevant food trends

Both hierarchical and k-means methods divided the MSs in the same clusters. Hierarchical agglomerative method was performed applying "complete" linkage to matrix of dissimilarities calculated with Euclidean distance, as producing the best separation of clusters. The clusters are visualised in Figure SM 2.2 in a bi-dimensional space defined by principal components (PCs). PCs are linear combinations of original variables

(i.e. the food consumption trends) (Abdi & Williams, 2010). The visualisation in Figure SM 2.2 is representative for both methods, because they produced the same clusters.

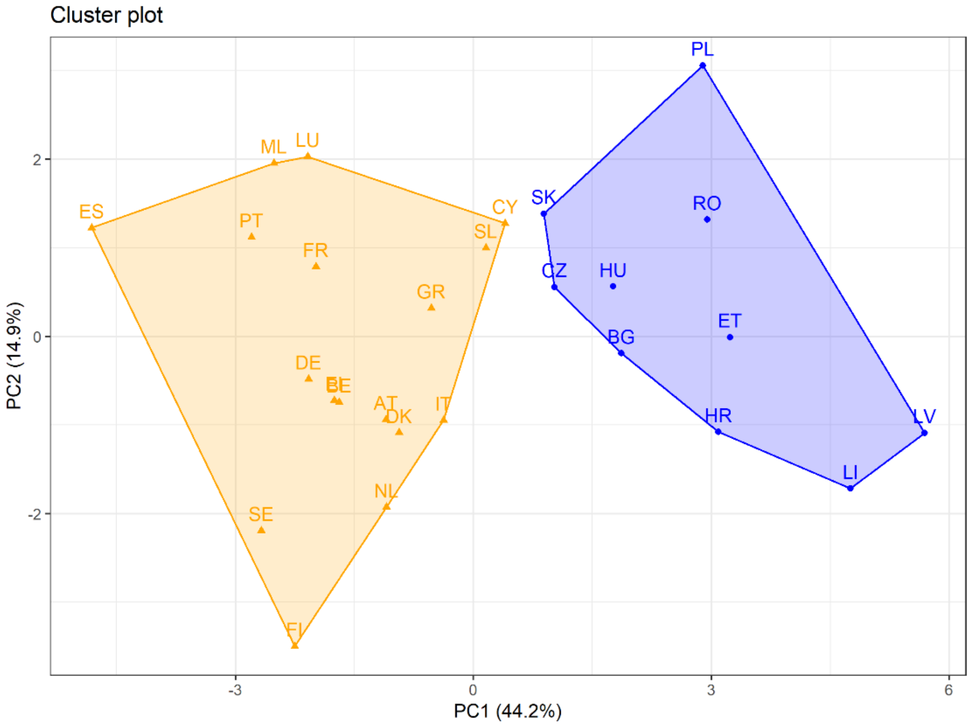

Figure SM 2.2. Clusters visualisation over Principal Components.

PCA is an extremely useful tool to explore variables contribution in the definition of clusters. PC1 (i.e., the x-axis in Figure SM 2.2) explains 44.2% of the total variability. It means the PC1 alone contains almost half of the overall information. Figure SM 2.2 shows that cluster separation occurs along PC1 (i.e., horizontal separation of the two groups), meaning that variables relevant to PC1 are those responsible for the division in clusters. On the contrary, the second PC (i.e., the y-axis) explains 14.9% of total variability and it can be used to understand intra-cluster variability (vertical distances among MSs).

Figure SM 2.3 reports the contribution of each variable to PC dimensions. All variables above the red dashed threshold are considered relevant. The red line represents the scenario with all variables equally contributing to the PC (i.e., 100% / number of variables = 100%/15 = 6.6%).

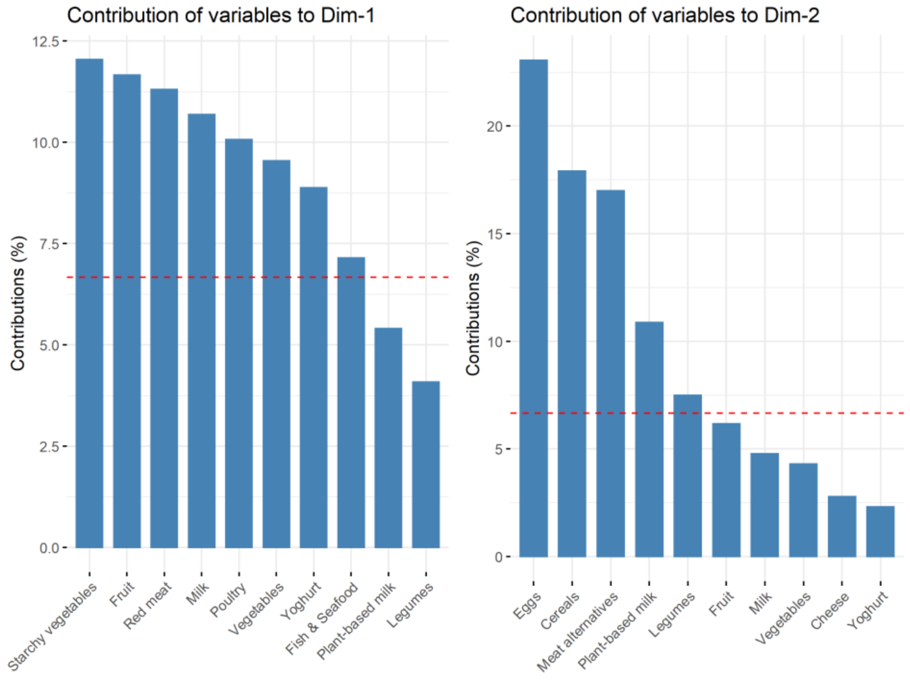

Figure SM 2.3. Variables contribution to Principal Components.

Trends of starchy vegetables, fruit, red meat, milk, poultry, vegetables, yoghurt, and fish & seafood are relevant for the first PC dimension, and thus for the definition of the clusters. Analysing the trends (Figure 2 of the manuscript), it could be noticed that increases in such variables are translated into positive PC1 values, and vice versa. For starchy vegetables, red meat, milk, yoghurt, and fish & seafood it is observed a common increasing trend in the blue cluster while decreasing in the orange cluster. Fruit, poultry, and vegetables are increasing in most of MSs, with faster rates for MSs in the blue cluster.

Eggs, cereals, meat alternatives, plant-based milk, and legumes are relevant variables to PC2, they can explain part of the intra-cluster variability. For example, in Figure SM 2.2, Poland (PL) is vertically very distant from the other countries belonging to the blue cluster. The rationale for this gap is the huge difference in trend of eggs. Indeed, eggs consumption decreased significantly (-1.8%) for Poland whereas it increased (+0.5% in average) in other MSs assigned to the same cluster. Moreover, the consumption of meat alternatives (+165.3%) is increasing in Poland more rapidly than other MSs (+21.7 in average).

Sensitivity analysis

Two sensitivity analysis were performed in order to evaluate the robustness of clusters definition.

The first was carried out by repeating the clustering process and removing one variable at time. Table SM 2.1 shows the countries differently classified in each round of the sensitivity analysis. The smaller the number of countries the more robust the results. Generally, k-means appear to be more stable compared to the hierarchical method. The stability is due to the presence of several relevant trends showing the same pattern. The difference in k-means classification regards Cyprus and Slovenia, which are the MSs belonging to the blue cluster closer to the orange cluster in Figure SM 2.2. Clusters visualisation over Principal Components. Hierarchical clustering is more sensitive to poultry, cheese and eggs variables, whose removals would cause the largest difference in classification. Cyprus, Slovakia, and Czech Republic are the countries most frequently classified in a different cluster, with the hierarchical method.

	No Fruits	No Nuts	No Vegetables	No starchy vegetables	No Cereals	No Red meat	No poultry	No Fish & seafood	No Meat alternatives	No legumes	No milk	No plant-based milk	No Cheese	No Yoghurt	No Eggs
**Hierarchical**		CY, SI			CZ, SI	CZ, SI	BG, CZ, HU, PL, RO, SK				SI		CY, EL, IT, SI	SI	CY, EL, IT, SI
**K-means**	CY, SI		CY, SI			CY, SI					CY, SI			CY	

Table SM 2.1. Sensitivity analysis. Columns names indicates the removed variable. Blank boxes mean no variation compared to the original result. Country codes indicates which MSs are classified in a different cluster

The second sensitivity analysis was performed to evaluate to which extent the modelled food items influence the cluster assignments. Table SM2.2 reports the share of modelled items per food category for the MSs with at least one modelled data. Modelled data are provided for eight MSs (i.e., Croatia, Cyprus, Estonia, Latvia, Lithuania, Luxembourg, Malta, and Slovenia), with three of them (i.e., Cyprus, Luxembourg, and Malta) for which all provided data are modelled.

	**Product category**	**Croatia**	**Cyprus**	**Estonia**	**Latvia**	**Lithuania**	**Luxembourg**	**Malta**	**Slovenia**
Major food groups	Cereals	91%	100%	91%	91%	91%	100%	100%	91%
	Cheese	100%	100%	100%	100%	100%	100%	100%	100%
	Eggs	100%	100%	100%	100%	100%	100%	100%	100%
	Fish & Seafood	100%	100%	100%	100%	100%	100%	100%	100%
	Fruit	100%	100%	100%	100%	100%	100%	100%	100%
	Legumes	100%	100%	100%	100%	100%	100%	100%	100%
	Meat alternatives	75%	100%	75%	75%	75%	100%	100%	75%
	Milk	86%	100%	86%	86%	86%	100%	100%	86%
	Nuts	100%	100%	100%	100%	100%	100%	100%	100%
	Plant-based milk	100%	100%	100%	100%	100%	100%	100%	100%
	Poultry	100%	100%	100%	100%	100%	100%	100%	100%
	Red meat	100%	100%	100%	100%	100%	100%	100%	100%
	Starchy vegetables	100%	100%	100%	100%	100%	100%	100%	100%
	Vegetables	100%	100%	100%	100%	100%	100%	100%	100%
	Yoghurt	0%	100%	0%	0%	0%	100%	100%	0%
Discretionary foods	Alcoholic drinks	0%	100%	0%	0%	0%	100%	100%	0%
	Cakes	0%	100%	0%	0%	0%	100%	100%	0%
	Confectionery	100%	100%	100%	100%	100%	100%	100%	100%
	Frozen desserts	100%	100%	100%	100%	100%	100%	100%	100%
	Juice	0%	100%	0%	0%	0%	100%	100%	0%
	Pastries	0%	100%	0%	0%	0%	100%	100%	0%
	Savoury snacks	100%	100%	100%	100%	100%	100%	100%	100%
	Soft drinks	17%	100%	17%	17%	17%	100%	100%	17%

	Sweet biscuits	0%	100%	0%	0%	0%	100%	100%	0%

Table SM2.2. Share of modelled food items per food category. Only MSs with at least one modelled food item are reported.

The sensitivity analysis was carried out by repeating the clustering in two scenarios: (i) EU24, excluding MSs with all modelled food categories; and (ii) EU19, excluding MSs with at least one modelled food item. Table SM2.3 compares the results of cluster analysis on EU27, with the ones of the two scenarios (i.e., EU24 and EU19), for both clustering methods. Results show that both hierarchical and k-means approaches produce the same partitioning as of EU27, in the exception of the excluded countries. The analysis on EU19 shows that only k-means is able to generate the same clusters of EU27 (a part from excluded MSs), and thus capture the different dietary trends described in the manuscript. On the contrary, hierarchical clustering produces a totally different division not able to capture relevant differences in dietary trends.

	**EU27 (2020 composition)**		**EU24**		**EU19**	
**Country**	**Hierarchical**	**K-means**	**Hierarchical**	**K-means**	**Hierarchical**	**K-means**
Austria	Orange	Orange	Orange	Orange	Blue	Orange
Belgium	Orange	Orange	Orange	Orange	Blue	Orange
Bulgaria	Blue	Blue	Blue	Blue	Blue	Blue
Croatia	Blue	Blue	Blue	Blue		
Cyprus	Orange	Orange				
Czech Republic	Blue	Blue	Blue	Blue	Blue	Blue
Denmark	Orange	Orange	Orange	Orange	Blue	Orange
Estonia	Blue	Blue	Blue	Blue		
Finland	Orange	Orange	Orange	Orange	Orange	Orange
France	Orange	Orange	Orange	Orange	Blue	Orange
Germany	Orange	Orange	Orange	Orange	Blue	Orange
Greece	Orange	Orange	Orange	Orange	Blue	Orange
Hungary	Blue	Blue	Blue	Blue	Blue	Blue
Ireland	Orange	Orange	Orange	Orange	Blue	Orange
Italy	Orange	Orange	Orange	Orange	Blue	Orange
Latvia	Blue	Blue	Blue	Blue		
Lithuania	Blue	Blue	Blue	Blue		
Luxembourg	Orange	Orange				
Malta	Orange	Orange				
Netherlands	Orange	Orange	Orange	Orange	Blue	Orange
Poland	Blue	Blue	Blue	Blue	Blue	Blue
Portugal	Orange	Orange	Orange	Orange	Orange	Orange
Romania	Blue	Blue	Blue	Blue	Blue	Blue
Slovakia	Blue	Blue	Blue	Blue	Blue	Blue
Slovenia	Orange	Orange	Orange	Orange		
Spain	Orange	Orange	Orange	Orange	Orange	Orange
Sweden	Orange	Orange	Orange	Orange	Orange	Orange

Table SM2.3. Comparison between clustering results on EU27 (2020 composition), EU24, and EU19, for both clustering methods.

Overall, the clusters assignment proposed in the paper is robust as two different approaches produced the same grouping. Additionally, the sensitivity analysis shows that the classification of Cyprus, Slovakia, and Czech Republic present a higher degree of uncertainty compared to the rest of MSs. Moreover, the presence of modelled food items is not influencing the results, as the same clusters are produced when MSs with modelled food items are excluded. Lastly, when several MSs are excluded from the analysis, the k-means approach performs better than the hierarchical method in capturing the relevant dietary changes.

3. Clusters' profiles

The profile illustrates the sales evolution of sales over time, it is representative of the behaviour of all MSs assigned to the same cluster. The consumption per capita is calculated as average of consumption of all MSs in the cluster, weighted on their population size. The "a" plots illustrate consumption per capita over years, while the "b" plots reports the percentage variation compared with the first year of the time series (i.e., 2008).

Major food groups

For a better comparison, product categories were reported in several figures: cereals, fruit, starchy vegetables, and vegetables in Figure SM 3.1; fish & seafood, milk, red meat, and poultry in Figure SM 3.2; legumes, meat alternatives, nuts, and plant-based milk in Figure SM 3.3; and cheese, eggs, and yoghurt in Figure SM 3.4. Each figure visualises product categories with similar consumption amounts.

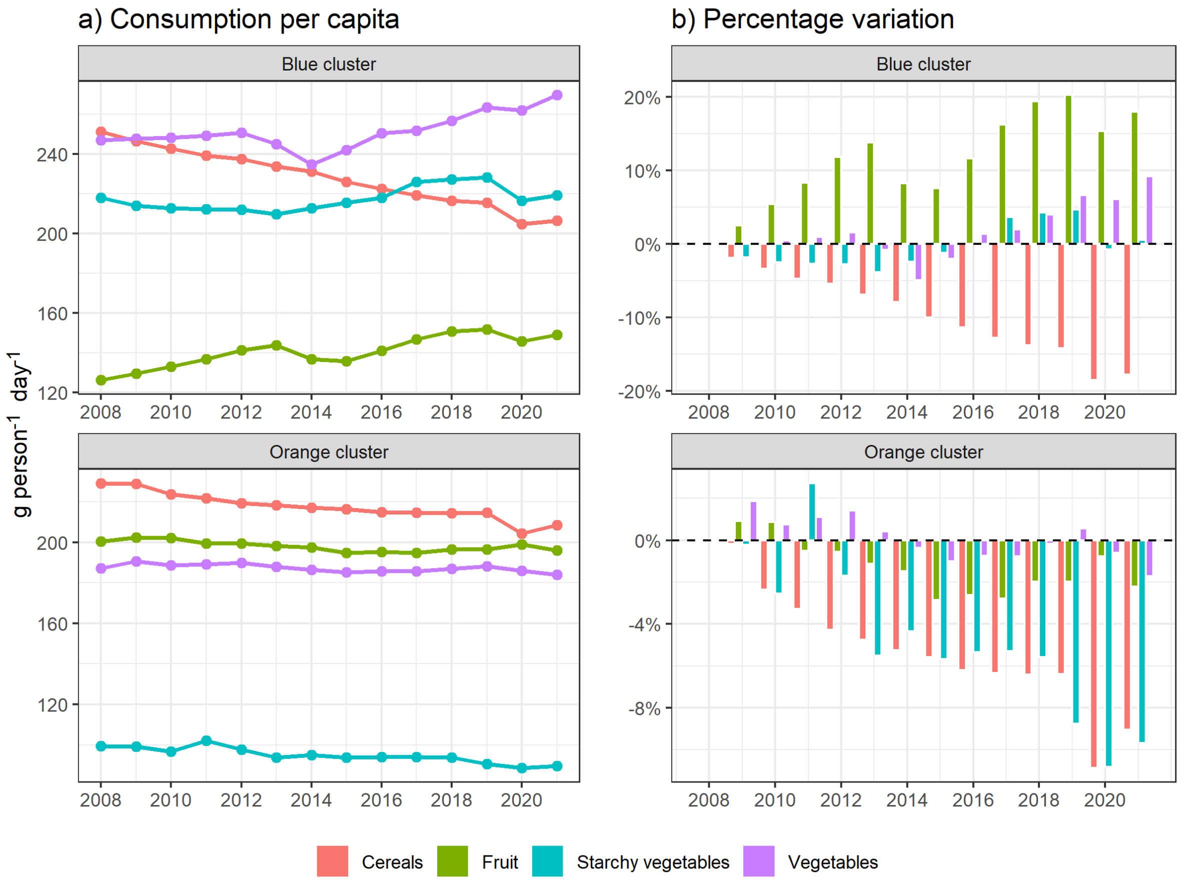

Figure SM 3.1. Average trends and percentage variation of sales of cereals, fruit, starchy vegetables, and vegetables, in the blue and orange clusters.

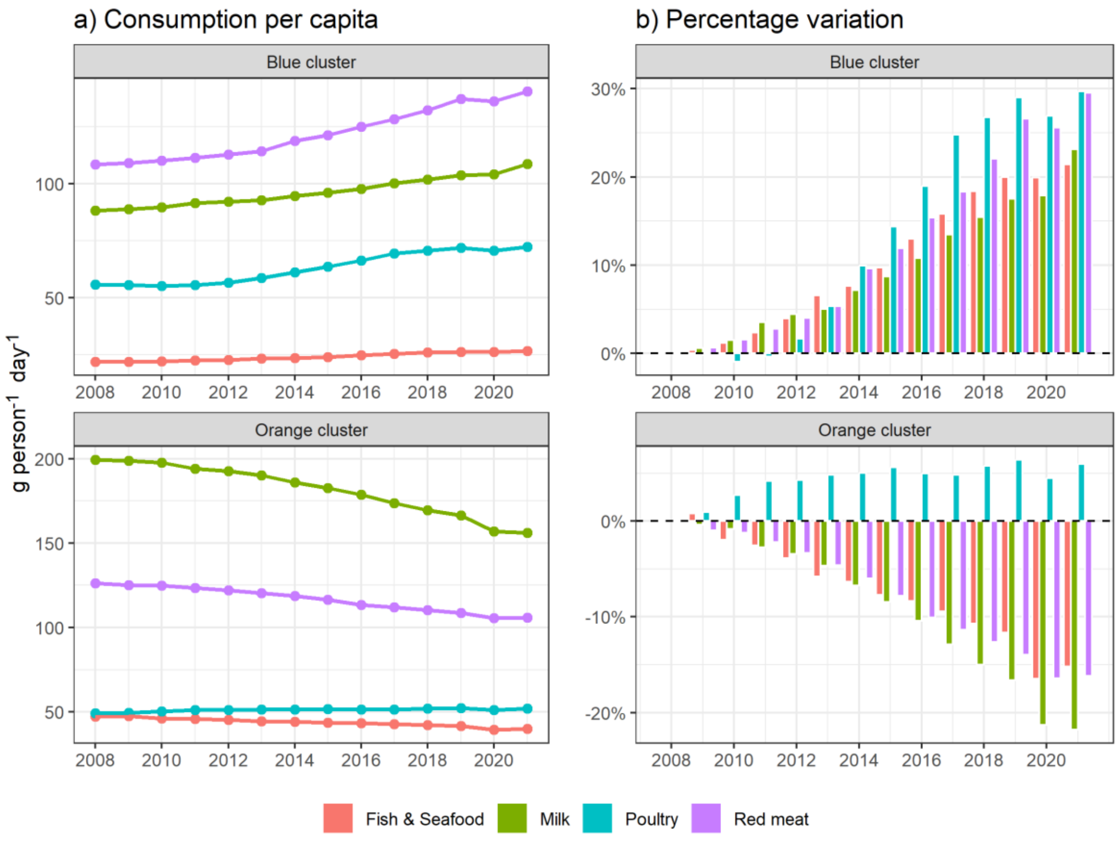

Figure SM 3.2. Average trends and percentage variation of trends of fish & seafood, milk, red meat, and poultry, in the blue and orange clusters.

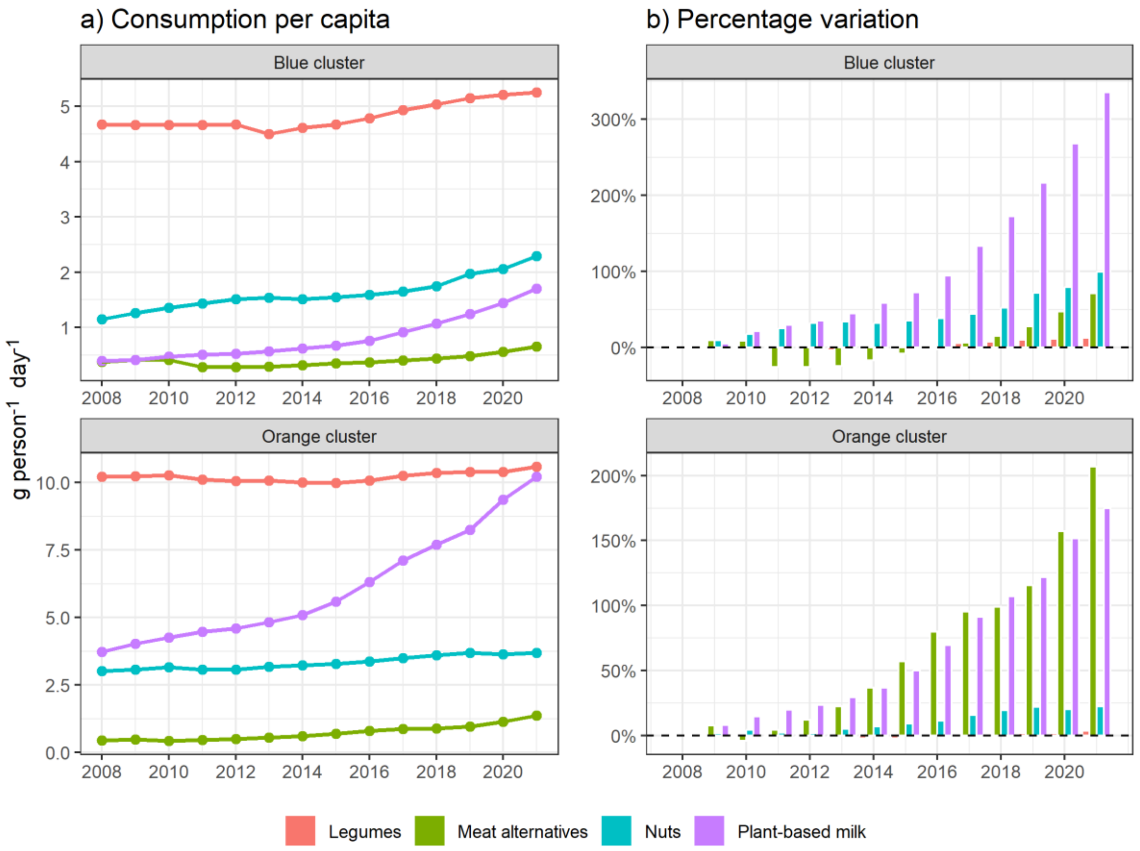

Figure SM 3.3. Average trends and percentage variation of sales of legumes, meat alternatives, nuts, and plant-based milk, in the blue and orange clusters.

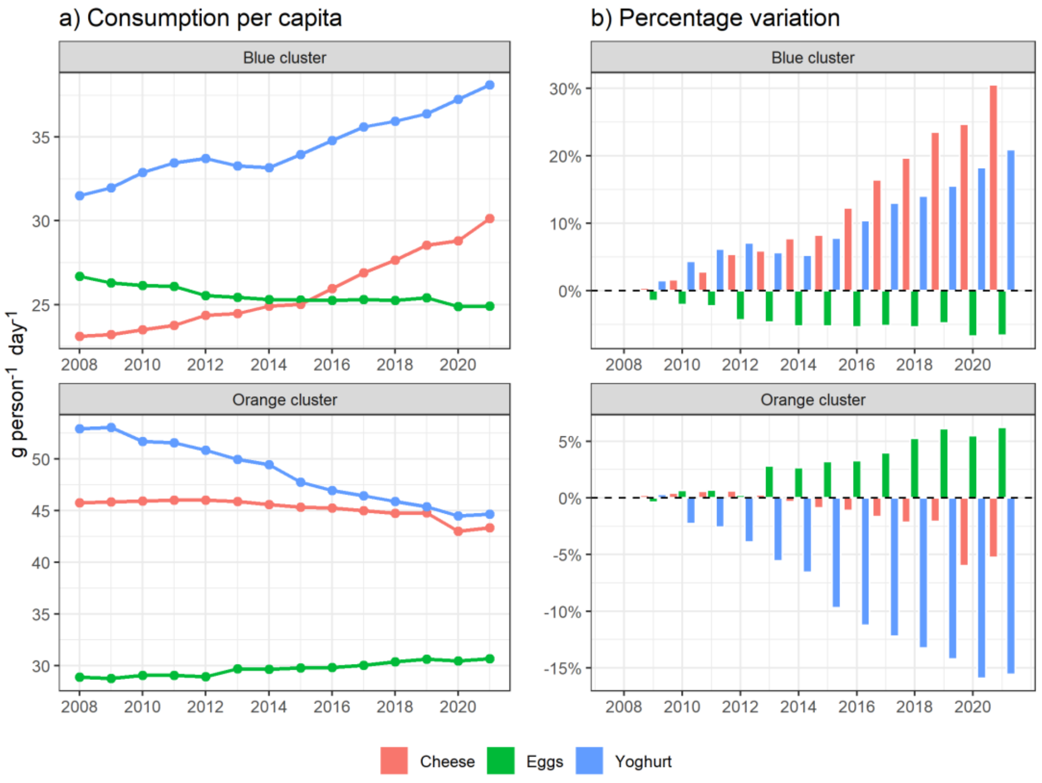

Figure SM 3.4. Average trends and percentage variation of sales of cheese, eggs, and yoghurt, in the blue and orange clusters.

Discretionary products

Profiles for alcoholic drinks, juice, and soft drinks are reported in Figure SM 3.5. Profiles for confectionery, frozen desserts, and savoury are reported in Figure SM 3.6. Profiles for cakes, pastries, and sweet biscuits are reported in Figure SM 3.7.

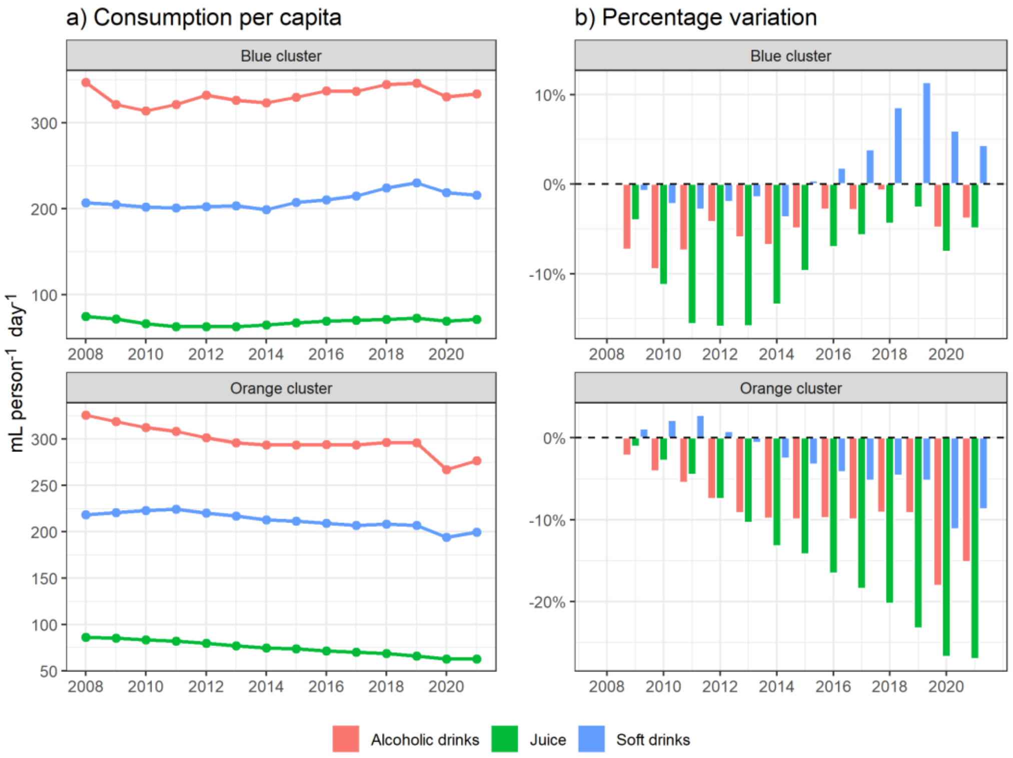

Figure SM 3.5. Average trends and percentage variation of sales of alcoholic drinks, juice, and soft drinks, in the blue and orange clusters.

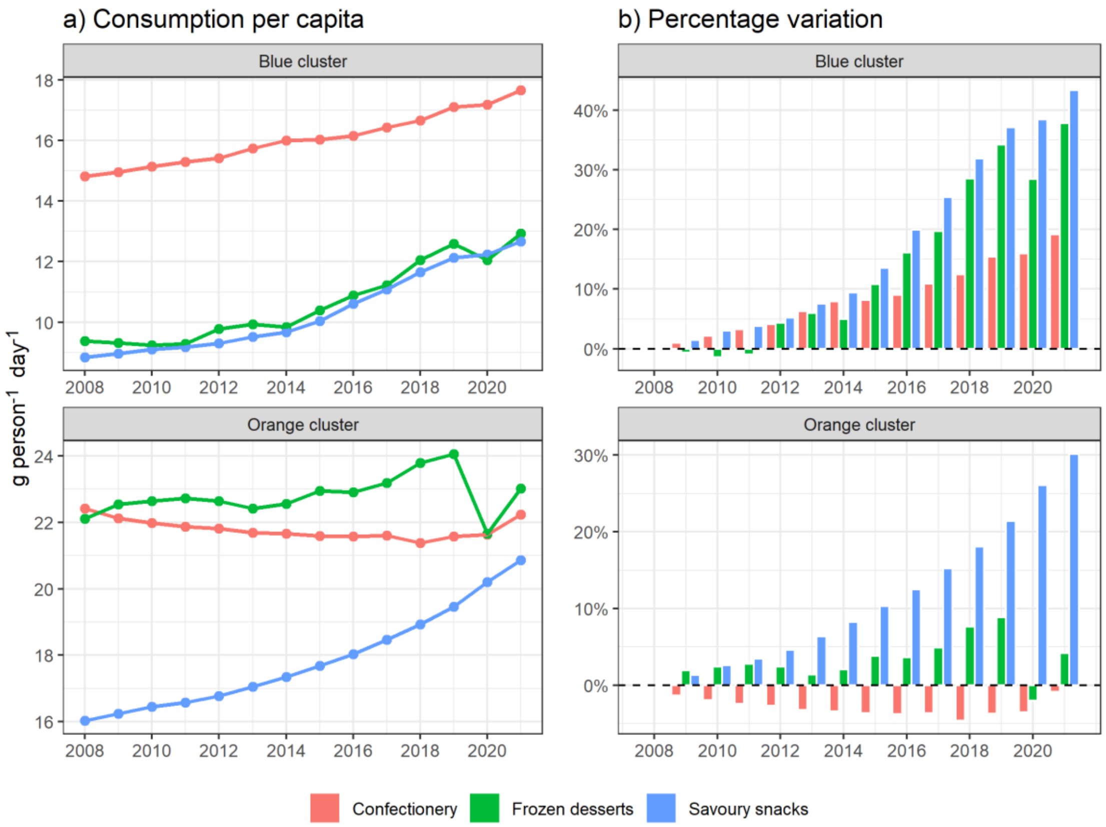

Figure SM 3.6. Average trends and percentage variation of sales of confectionery, frozen desserts, and savoury, in the blue and orange clusters.

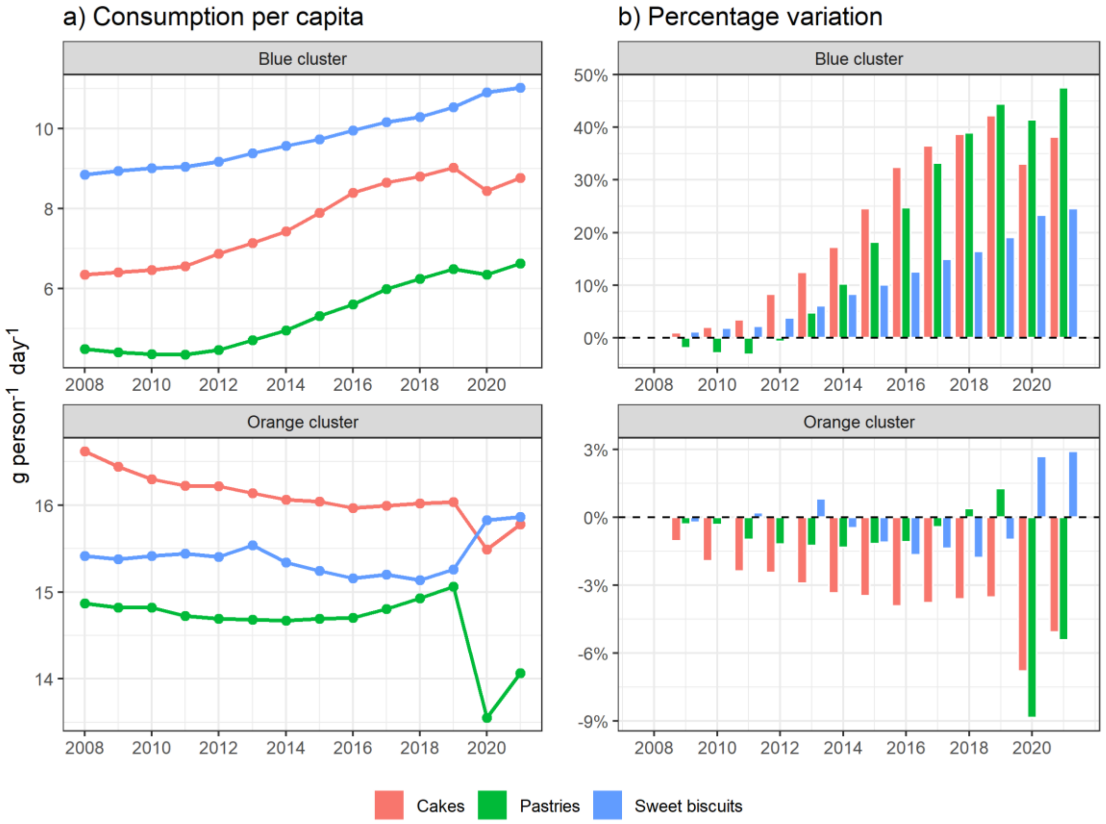

Figure SM 3.7. Average trends and percentage variation of sales of cakes, pastries, and sweet biscuits, in the blue and orange clusters.

Fresh and processed fruit, vegetables and starchy vegetables

Profiles for fresh fruit, fresh starchy vegetables, and fresh vegetables are reported in Figure SM 3.8. Profiles for processed fruit, processed starchy vegetables, and processed vegetables are reported in Figure SM 3.9.

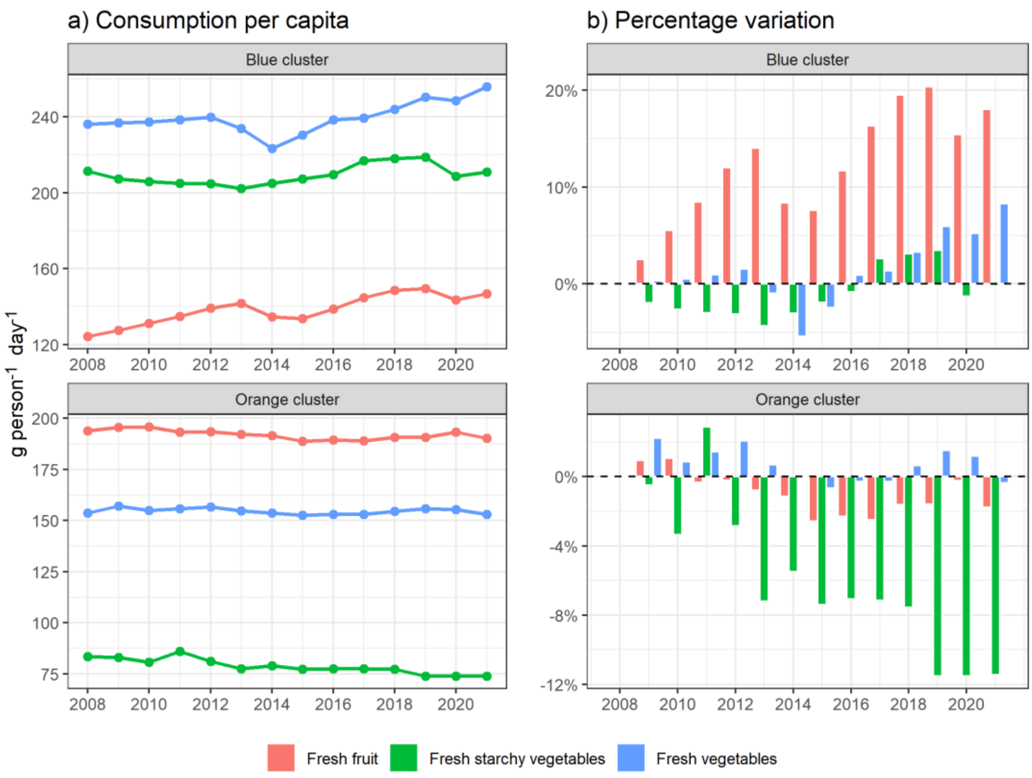

Figure SM 3.8. Average trends and percentage variation of sales of fresh fruit, fresh starchy vegetables, and fresh vegetables, in the blue and orange clusters.

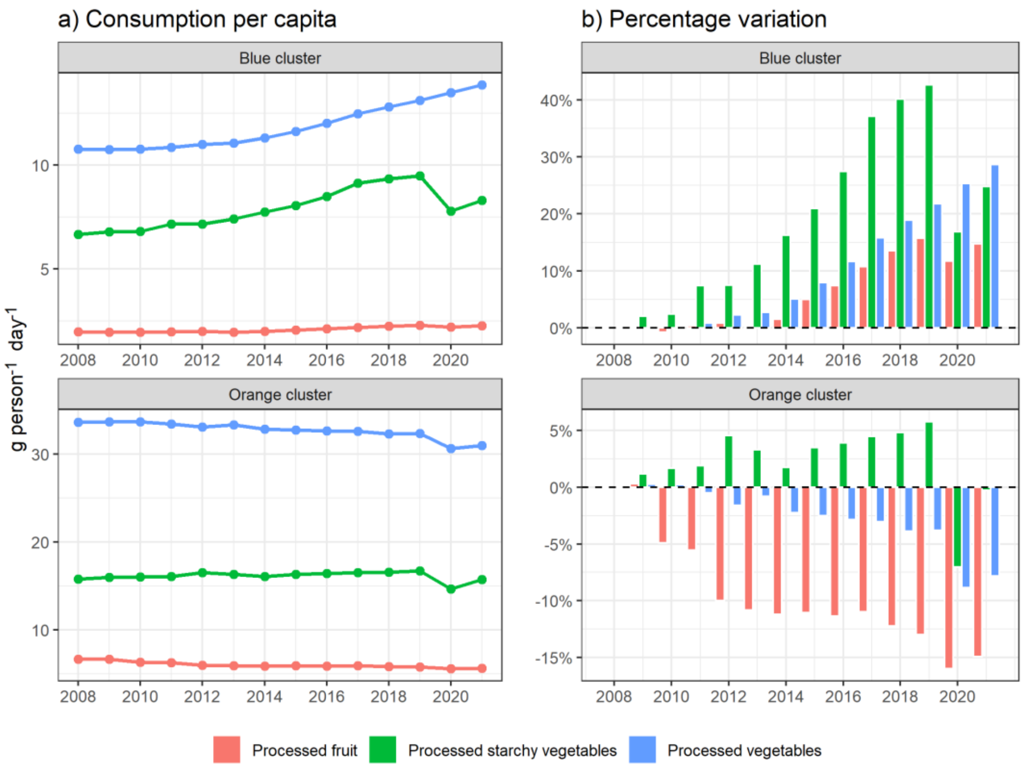

Figure SM 3.9. Average trends and percentage variation of sales of processed fruit, processed starchy vegetables, and processed vegetables, in the blue and orange clusters.

4. Comparison with attitudinal surveys

Trends of meat consumption and its substitutes were further compared with results collected through a Eurobarometer survey measuring Europeans' attitudes to take action against climate change. Eurobarometer regularly monitors the state of public opinion in the EU. Roughly 1000 participants per MS took part in the survey, systematically recruited from different social and demographic categories. Participants were asked "*which of the following actions have you personally taken to fight climate change over the past six months?*" (question QB6) with "*Buy and eat less meat*" among the options available.

Figure SM4.1 compares the survey results with consumption trends of red meat, poultry and meat alternatives. The x-axis reports the average yearly increase, while the y-axis lists the MSs grouped per cluster and ordered from greatest to lowest share (in brackets) of people that reported to *eat less meat*.

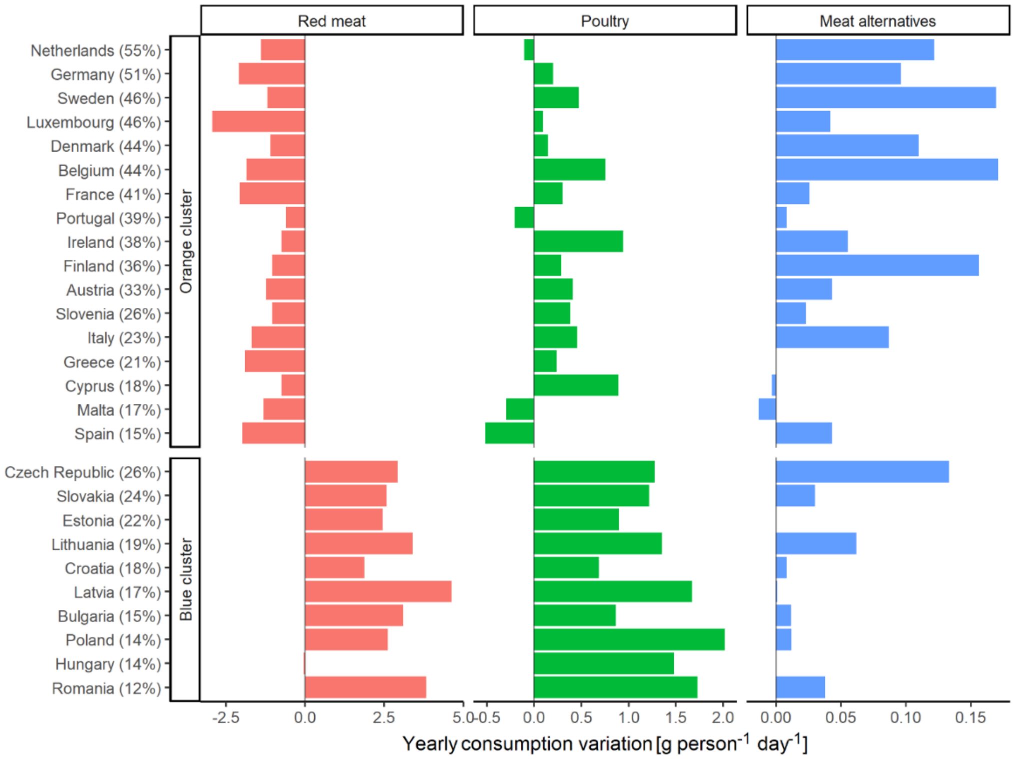

Figure SM4.1. Consumption trends of red meat, poultry and meat alternatives, grouped per cluster and listed per attitude to eat less meat. In brackets, the share of people reporting to eat less meat in each MS. Countries grouped per clusters as defined for the major food groups (Figure 1 of the main text). Please note that the scale of the x-axis is different for red meat, poultry and meat alternatives.

At the top, MSs with higher stated attitude towards eating less meat in the population show reduction of red meat consumption and increase of meat alternatives consumption. On the contrary, MSs with population stating to be less willing to *eat less meat* (at the bottom) are increasing the consumption of red meat. For Italy, Greece, Cyprus, Malta and Spain a reduction of red meat consumption was observed despite registering low attitude to *eat less meat* (23%, 21%, 18%, 17% and 15%, respectively), entailing that other factors than climate awareness could be driving their trends. Poultry consumption is increasing in almost all MSs, with faster increase in countries less willing to consume less meat.

In the Netherlands 55% of survey sample declared they are eating less meat. Indeed, this is reflected in the observed decreasing consumption trend of red meat and (to a lower extent) poultry together with an increasing trend of consumption of meat alternatives. On the other hand, Romania reports the lowest percentage of people with the attitude in reducing meat intake (12%) and opposite trends: strong increase of red meat consumption, moderate increase of poultry and slight increase of meat alternatives intake.

References

Abdi H., Williams, L.J., 2010. Principal component analysis. Wiley interdisciplinary reviews: computational statistics, 2(4), 433-459.

Charrad M., Ghazzali N., Boiteau V., Niknafs A., 2014. NbClust: An R Package for Determining the Relevant Number of Clusters in a Data Set. Journal of Statistical Software, 61(6), 1–36. https://doi.org/10.18637/jss.v061.i06

Li, G., 2006. Robust Regression. In Exploring Data Tables, Trends, and Shapes (eds D.C. Hoaglin, F. Mosteller and J.W. Tukey). https://doi.org/10.1002/9781118150702.ch8

Huber, P.J., 2011. Robust Statistics. In: Lovric, M. (eds) International Encyclopedia of Statistical Science. Springer, Berlin, Heidelberg. https://doi.org/10.1007/978-3-642-04898-2_594

Rousseeuw P.J., 1987. Silhouettes: a Graphical Aid to the Interpretation and Validation of Cluster Analysis. Computational and Applied Mathematics. 20: 53–65. doi:10.1016/0377-0427(87)90125-7.

Tibshirani R., Walther G., Hastie T., 2001. Estimating the number of clusters in a data set via the gap statistic. Journal of the Royal Statistical Society, Series B. 63: 411–423. doi:10.1111/1467-9868.00293.

Thorndike R.L., 1953. Who Belongs in the Family?. Psychometrika. 18 (4): 267–276. doi:10.1007/BF02289263
